# Beyond monoclonal antibodies: constraints and the case for alternative PD-1/PD-L1-targeting formats

**DOI:** 10.3389/fimmu.2025.1729468

**Published:** 2025-12-17

**Authors:** Natalia O. Porozova, Natalia V. Petukhova, Maxim A. Gureev, Ivan S. Moiseev, Igor E. Eliseev, Ludmila V. Fedorova, Yuri B. Porozov

**Affiliations:** 1 Laboratory of Bio- and Chemoinformatics, Faculty of Computer Science, Physics and Technology, National Research University Higher School of Economics, St. Petersburg, Russia; 2 Bioinformatics Research Center, Pavlov First Saint Petersburg State Medical University, Saint Petersburg, Russia; 3 Raisa Maximovna Gorbacheva Research Institute, Pavlov First Saint Petersburg State Medical University, St. Petersburg, Russia; 4 Advitam Laboratory, Belgrade, Serbia

**Keywords:** bispecific antibodies, decoy receptors, Fc engineering, intratumoral delivery, PD-1, PD-L1, reduced-size antibody scaffolds, small molecules

## Abstract

Programmed cell death protein 1 (PD-1)/programmed death-ligand 1 (PD-L1) blockade has reshaped immuno-oncology, with antibodies establishing benchmarks for survival and durability of response across multiple tumor types. Yet format-related constraints of monoclonal antibodies persist, including incomplete and heterogeneous intratumoral penetration, long half-life with limited real-time toxicity control, immune-related toxicities, and context-dependent resistance within the tumor microenvironment. This Review synthesizes the current landscape and near-term prospects of complementary or alternative modalities for PD-1/PD-L1 pathway control. Within antibody-based approaches, we first consider clinical combinations of classical PD-1/PD-L1 antibodies and then review bispecific antibodies that integrate blockade with co-inhibition or context-dependent co-stimulation, reduced-size antibody scaffolds with tunable pharmacokinetics and diffusion, Fc-engineered formats to modulate effector functions, and locally delivered or gene-encoded fragments that intensify intratumoral exposure. Beyond conventional antibodies, we appraise non-antibody biologics and small-molecule agents, including small molecules that promote PD-L1 dimerization to occlude the PD-1 interface, as well as decoy receptors and aptamers, and delivery-focused strategies that enable spatially constrained or vectorized pathway modulation. Across these modalities, we emphasize fit-for-context design that links target biology to the physicochemical profile of each format and route of administration, and we consolidate evidence from preclinical to late-phase settings. Building on prior reviews that have cataloged clinical outcomes of PD-1/PD-L1 antibodies and provided in-depth overviews of individual emerging modalities, this article adopts a format-centered, cross-modality conceptual framework that connects antibody-intrinsic and microenvironmental constraints to the design and emerging clinical positioning of alternative PD-1/PD-L1-targeting modalities, to clarify when and how intensification or format switching is most likely to be beneficial. We conclude that PD-1/PD-L1 antibodies retain a foundational role, while additional modalities are positioned to serve as complementary or alternative options that mitigate inherent physical and biological limitations when matched to the spatial, cellular, and pharmacological constraints of individual tumors.

## 1 Introduction

Programmed cell death protein 1 (PD-1), a member of the immunoglobulin superfamily, is induced on T cells and other immune populations upon activation and serves as a negative regulator that prevents excessive stimulation and autoimmunity (1). PD-1 engages two physiological ligands, PD-L1 and PD-L2, which are inhibitory B7 family members expressed on diverse cell types (2–4). On antigen-presenting cells, PD-L1 can be induced by innate and cytokine signals, whereas on T and B lymphocytes it increases following T-cell receptor (TCR) activation (2). In tumors, PD-L1 is upregulated by interferon-γ and other inflammatory cues, contributing to immune evasion; sustained antigenic stimulation drives PD-1 expression and an exhausted T-cell phenotype (5, 6). Circulating soluble PD-L1 has been associated with poor responsiveness to PD-1/PD-L1 blockade, although its biology remains incompletely defined (1).

Ligand engagement inhibits TCR/CD28 signaling and dampens effector function, enabling tumors to suppress priming and cytotoxic elimination (7). Recognition of this axis more than three decades ago set the stage for immune-checkpoint therapies that have delivered substantial clinical benefit in selected tumor types, while also revealing class-specific limitations and immune-related toxicities (6, 8, 9). As longer-term clinical and real-world data accumulate, including 10-year follow-up with nivolumab plus ipilimumab in advanced melanoma and landmark safety analyses with pembrolizumab (10, 11), it is increasingly clear that, despite their transformative role, PD-1/PD-L1 antibodies deliver durable benefit only for a subset of patients and are constrained by immune-related toxicities, organ-specific barriers, and the practical burden of prolonged intravenous treatment. In parallel, bispecific and reduced-size antibody formats and first-in-class non-antibody PD-1/PD-L1 modulators have reached regulatory approval or late-phase evaluation in selected indications, making questions of molecular format and delivery route an immediate rather than hypothetical consideration (12).

These developments provide a rationale for a format-focused reappraisal of PD-1/PD-L1 pathway control (13). Against this backdrop, the present review concentrates on format-driven strategies to refine PD-1/PD-L1 blockade. Within antibody-based approaches, clinical combinations of classical PD-1/PD-L1 antibodies have expanded to include CTLA-4 co-blockade, chemoimmunotherapy, targeted agents and myeloid modulators, while in parallel bispecific antibodies that integrate blockade with co-inhibition or context-dependent co-stimulation, reduced-size antibody scaffolds with tunable pharmacokinetics and diffusion, Fc-engineered formats to modulate effector functions and locally delivered or gene-encoded fragments that intensify intratumoral exposure are moving through preclinical and clinical evaluation.

Beyond conventional antibodies, non-antibody biologics and small-molecule agents are being actively developed, including small molecules that promote PD-L1 dimerization to occlude the PD-1 interface, as well as decoy receptors, aptamers and delivery-focused strategies that enable spatially constrained or vectorized pathway modulation (14–16). Prior reviews have typically considered these strands separately, focusing recent landscape and format-focused analyses as examples (17), and only occasionally treating the molecular format itself as the organizing principle for understanding limitations and design options. Here, the goal of this review is to provide a concise, format-centered framework that links the physical and pharmacologic constraints of PD-1/PD-L1 antibodies to the properties, delivery routes and near-term clinical positioning of alternative modalities, so that readers can judge when antibody-based intensification may be sufficient and when alternative formats may reasonably be explored in specific biological and clinical contexts. For the reader’s convenience, abbreviations that appear in the text are also defined in the glossary (**Supplementary Table S1**).

## 2 PD-1/PD-L1 axis: structure, ligands and signaling mechanisms

### 2.1 PD-1: structure, expression and functional properties

**Figure 1:**
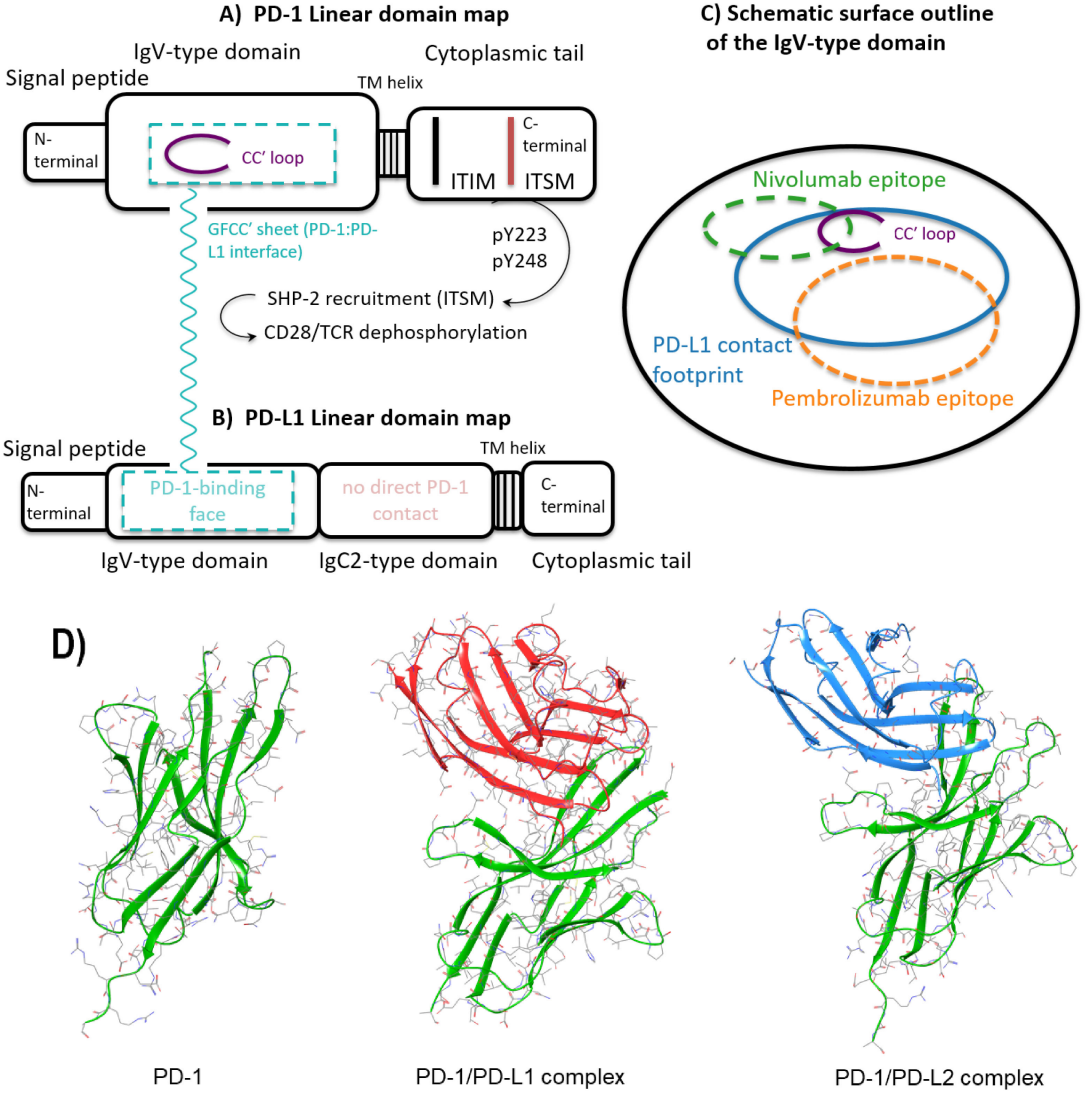
Domain architecture and signaling motifs (contact regions) of PD-1 and PD-L1. **(A)** Linear domain map of human PD-1 showing the signal peptide, an extracellular IgV-type domain, a single-pass transmembrane helix and a cytoplasmic tail with ITIM and ITSM motifs; the CC′ loop and GFCC′ β-sheet are highlighted as the PD-L1 interaction surface, with arrows indicating downstream SHP-2 recruitment and CD28/TCR dephosphorylation. **(B)** Linear domain map of human PD-L1 with an N-terminal IgV-type domain labeled as the PD-1-binding face, followed by an IgC2-type domain, a transmembrane helix and a short cytoplasmic tail; the membrane-proximal region and cytoplasmic tail are annotated as having no direct PD-1 contact. **(C)** Schematic surface outline of the PD-1 IgV-type domain showing the PD-L1 contact footprint and the partially overlapping epitopes of nivolumab and pembrolizumab around the CC′ loop. **(D)** The β-sheet structures of PD-1 (PDB 3RRQ) and its complex with ligands PD-L1 (PDB 6UMT) and PD-L2 (PDB 4ZQK). The structures of PD-1 (PDB 3RRQ) and its complex with ligands PD-L1 (PDB 6UMT) and PD-L2 (PDB 4ZQK). Ribbon view comparing PD-1 alone (PDB 3RRQ) with its co-crystal complexes bound to PD-L1 (6UMT) and PD-L2 (4ZQK), highlighting the conserved IgV docking surface on PD-1. The figure shows that PD-L1 and PD-L2 contact overlapping epitopes with similar geometry, illustrating the shared receptor–ligand interface underlying checkpoint signaling. Further mechanistic explanation and references are provided in the main text. TM helix, transmembrane helix; CC′ loop/GFCC′ sheet, surface-exposed loop and β-sheet elements within the PD-1 IgV-like domain that form the PD-L1/antibody binding interface; ITIM, immunoreceptor tyrosine-based inhibitory motif; ITSM, immunoreceptor tyrosine-based switch motif; SHP-2, Src homology 2 (SH2) domain-containing phosphatase 2; TCR, T cell receptor; CD28, co-stimulatory receptor CD28 on T cells.

PD-1 is a member of the immunoglobulin superfamily induced on activated immune cells and functions as a negative regulator that prevents excessive stimulation and autoimmunity (8). The protein comprises an extracellular IgV-type domain, a single transmembrane helix and a cytoplasmic tail that contains the immunoreceptor tyrosine-based inhibitory motif (ITIM) and the immunoreceptor tyrosine-based switch motif (ITSM) required for inhibitory signaling (18) (**Figure 1A**). PD-1 engages two physiological ligands, PD-L1 and PD-L2, which are inhibitory B7 family members expressed on diverse cell types (2, 3). On antigen-presenting cells, PD-L1 can be induced by lipopolysaccharide and GM-CSF, whereas on T and B lymphocytes it increases following T-cell receptor activation (2). In tumors, PD-L1 is upregulated by interferon-γ and other inflammatory cues from T cells, NK cells and myeloid cells, contributing to immune evasion (5, 19). PD-L2 expression is detected on subsets of dendritic cells and specific tumor cell lines (3, 20, 21). Soluble PD-L1 (sPD-L1) has been associated with poor responsiveness to PD-1/PD-L1 blockade, although its biology remains incompletely defined (22–24); see **Supplementary Table S2** for induction and cellular sources and sPD-L1 correlates.

The extracellular domain of PD−1 is formed by nine β−strands connected by eight loops, which adopt a β−sandwich Ig-like topology along with a flexible loop located at the N−terminus (25). It also features several N−glycosylation sites, which can influence the binding of specific antibodies (26).

### 2.2 PD-L1: structure and functional properties

PD-L1 (CD274) is a type I transmembrane B7 family ligand comprising an extracellular N-terminal IgV-type domain that forms the PD-1 binding interface, a C-terminal IgC2-type domain, a single-pass transmembrane helix, and a short cytoplasmic tail with no known signaling motifs (**Figure 1B**). Its expression is inducible by interferon-γ and inflammatory cues across antigen-presenting cells and tumor cells, and it can also be upregulated on activated lymphocytes. At the structural level, PD-1 engages PD-L1 in a 1:1 complex dominated by contacts on the PD-1 GFCC′ sheet and CC′ loop and the PD-L1 IgV face (27); biophysical studies indicate a moderate-affinity, entropy-favored interaction, whereas PD-1 binds PD-L2 with higher affinity and a larger enthalpic contribution (28). These ligand-specific differences, together with heterogeneous and inducible PD-L1 expression in tumors, shape the pharmacology of PD-1/PD-L1 blockade and contribute to context-dependent clinical effects. For induction patterns and sPD-L1 correlates see **Supplementary Table S2**. Representative PD-1:PD-L1 complex structures are listed in **Supplementary Table S4**, and the N-terminal IgV domain of PD-L1 follows the canonical nine-stranded β-sandwich fold with connecting loops (29).

### 2.3 PD-1:PD-L1 complex and ligand/antibody binding hotspot

Structural studies of PD-1 ectodomains and their complexes with ligands or antibodies, including apo PD-1 (3RRQ) and PD-1:PD-L1 complexes (6UMT, 4ZQK), show that the ectodomain adopts a two-sheet β-sandwich stabilized by a Cys54–Cys123 disulfide, and that ligand recognition centers on the GFCC′ sheet and CC′ loop (27, 30, 31). In these complexes, PD-1 and PD-L1 adopt a nearly orthogonal orientation, with an extended, predominantly hydrophobic interface supported by intermolecular hydrogen bonds and π–π stacking interactions that stabilize the PD-1–PD-L1 complex (27, 30). By contrast, PD-1 binds PD-L2 with higher affinity and a larger enthalpic component, reflecting additional contacts within the GFCC′ region (28) (see **Supplementary Table S3** for residue-level and conformational notes). From a therapeutic perspective, these structural data localize most ligand contacts and high-affinity antibody epitopes to the N-terminal segment, CC′ loop, and GFCC′ sheet, which together form a relatively compact hot spot for PD-1:ligand engagement and drug binding (**Figures 1C, D**).

### 2.4 Ligand engagement and therapeutic antibody blockade

Ligand engagement triggers inhibitory signaling by enabling TCR-proximal phosphorylation of ITIM and ITSM, recruitment and activation of Src-homology region 2-containing protein tyrosine phosphatase-2 (SHP-2) via its tandem Src homology 2 (SH2) domains and subsequent dephosphorylation of CD28/TCR pathway nodes, which attenuates PI3K–AKT and RAS–ERK signaling and suppresses T-cell effector function (32, 33). SHP-2 is generally considered the principal mediator of PD-1 inhibition, although context-dependent compensation by SHP-1 has been reported (34, 35); **Supplementary Table S4** lists the Protein Data Bank (PDB) accessions and primary article references. Because ITIM and ITSM are the primary conduits of PD-1-mediated inhibition, effective extracellular blockade needs to sufficiently disrupt ligand binding to prevent phosphorylation of these motifs and subsequent SHP-2 recruitment.

Therapeutic antibodies block PD-1 by binding the IgV-type domain and occluding the ligand interface that is centered on the N-terminal segment, CC′ loop, and GFCC′ sheet (**Figure 1C**). Nivolumab engages the N-terminal segment and CC′ loop, while pembrolizumab recognizes an extended epitope across the GFCC′ sheet, yielding near-complete coverage of the PD-L1-binding surface (26, 36, 37). By targeting these ligand-contacting regions, antibody binding stabilizes PD-1 in a state incompatible with ligand engagement, thereby preventing ITIM/ITSM-dependent signaling and releasing T-cell inhibition (30, 36).

Relative to small molecules, therapeutic antibodies and non-antibody formats differ in epitope coverage, binding kinetics, distribution and pharmacology; conclusions about superiority require context-specific data rather than general class assumptions (38–40).

## 3 Limits of PD-1/PD-L1 antibodies and the rationale for alternative scaffolds

Although mAbs are potent and highly specific PD-1 blockers, their clinical performance is constrained by three categories of limitation: immune-related toxicities and organ-specific safety concerns, pharmacokinetic and tissue-penetration barriers, and adaptive or microenvironmental resistance within the tumor and host immune system (41). We then consider how these constraints manifest with current PD-1/PD-L1 antibodies, then consider complementary PD-1/PD-L1-blocking strategies (for example, nanobodies and rationally designed small molecules) that may mitigate them.

### 3.1 Immune-related adverse events with PD-1/PD-L1 blockade (cardiovascular focus)

Immune-related adverse events (irAEs) are a major constraint of immune checkpoint inhibitor (ICI) therapy; PD-1/PD-L1 inhibitors are associated with a spectrum of cardiovascular irAEs, notably autoimmune myocarditis, although infrequent, carry disproportionate morbidity and mortality (42). Disinhibition of PD-1/PD-L1 and cytotoxic T-lymphocyte-associated protein 4 (CTLA-4) pathways breaks peripheral tolerance and can precipitate T-cell cross-reactivity against cardiac autoantigens (molecular mimicry) (43). In fatal cases with combined PD-1 and CTLA-4 blockade, TCR sequencing has demonstrated shared clonotypes in the tumor and myocardium, consistent with immune-mediated myocarditis (43). Preclinical evidence shows that loss of PD-1 or CTLA-4 signaling produces lethal myocarditis in mouse models (44, 45).

At the antigenic level, cardiac and vascular toxicity has been linked to T-cell responses against cardiomyocyte and endothelial antigens (46–51). Dysregulated cytokine signaling, particularly TNF-α, IFN-γ, IL-1β, IL-6 and IL-17, contributes to myocardial inflammation, endothelial activation/dysfunction and atherothrombotic risk (52–54). Emerging clinical and experimental data also support targeting the interleukin-6 (IL-6)-Janus kinase 2 (JAK2)-signal transducer and activator of transcription 3 (STAT3) axis (e.g., tocilizumab) in steroid-refractory cases (55–57). Multiple cohorts and imaging studies indicate an increased incidence of atherosclerotic cardiovascular events and measurable progression of vascular inflammation/plaques during ICI exposure, consistent with plaque destabilization and thrombotic complications in susceptible patients (58–61).

There are no established prophylactic strategies for immune-related adverse events (irAEs) induced by immune checkpoint inhibitors (ICIs). Interleukin-10 (IL-10), an immunoregulatory cytokine, has been proposed as a theoretical adjunct to PD-1/PD-L1 blockade to temper off-target inflammation while sustaining antitumor immunity; mechanistic and early clinical work with pegylated IL-10 (pegilodecakin) shows CD8+ T-cell activation and feasibility with anti-PD-1 therapy, yet randomized phase 2 trials in non-small-cell lung cancer did not improve efficacy and reported higher toxicity versus ICIs alone, so there is no guideline-endorsed prophylactic role (62, 63). Current guidelines emphasize reactive rather than preventive management of irAEs, with systemic glucocorticoids as first-line therapy once toxicities occur (64–66), and discourage routine glucocorticoid premedication except for patients with a documented history of infusion reactions (67).

Cardiotoxicity, most notably myocarditis, remains a safety bottleneck. Recent studies support early diagnostic pathways incorporating biomarkers and imaging, and suggest benefit from high-dose pulse glucocorticoids for confirmed ICI myocarditis (68, 69), alongside surveillance strategies to detect subclinical myocardial injury in high-risk groups (70–72).

### 3.2 Limited penetration of monoclonal antibodies into solid tumors

Monoclonal Abs exhibit limited penetration into solid tumors, which impedes achieving therapeutically effective concentrations in the tumor microenvironment (73–75). This limitation reflects a combination of the binding-site barrier, elevated interstitial fluid pressure and a dense extracellular matrix that together restrict macromolecular diffusion and retention (76, 77). Heterogeneous intratumoral distribution may contribute to treatment failure and the emergence of acquired resistance mechanisms (78).

Solid tumors commonly display poor vascularization and hypoxic niches that further compromise intratumoral distribution of PD-1/PD-L1 antibodies (79, 80). The binding-site barrier is particularly relevant, as antibodies rapidly engage PD-L1 at the tumor-immune interface, limiting penetration into deeper tumor regions (81, 82). In addition, PD-L1 expression within solid tumors is often heterogeneous and can be induced by interferon-γ (IFN-γ), creating immune-inflamed “hot spots” that sequester antibody binding and leave other tumor compartments underexposed (83–85).

By 2025, additional pharmacokinetic aspects of this limitation have been clarified. Anti-drug antibodies (ADA) against anti-PD-1 mAbs are increasingly recognized as contributors to accelerated clearance, reduced systemic exposure, and diminished response probability, supporting selective therapeutic monitoring of exposure and immunogenicity in patients with suspected early loss of drug activity (86, 87). Organ-specific barriers are particularly pronounced in central nervous system disease, where the blood-brain barrier limits mAb penetration and the need for steroids can further blunt benefit; early data on intrathecal anti-PD-1 administration for leptomeningeal metastases suggest feasibility and acceptable safety but rest on a limited evidentiary base that warrants prospective evaluation (88). From a delivery perspective, 10-minute nivolumab/pembrolizumab infusions and subcutaneous pembrolizumab (with hyaluronidase), which has shown non-inferior PK to intravenous dosing, may ease resource burden and expand access but do not overcom the fundamental diffusion and binding-site barriers that constrain antibody penetration into solid tumors (89–91).

### 3.3 Tumor immune evasion and microenvironmental resistance

*Malignant cells* employ multiple mechanisms to evade immune surveillance, of which PD-1/PD-L1 signaling constitutes only one axis of suppression (92). Notably, even tumors with a high tumor mutational burden (TMB) or microsatellite instability-high/deficient mismatch repair (MSI-H/dMMR) phenotype – features generally associated with heightened immunogenicity – can manifest primary or acquired resistance to PD-1 (92). Contributing mechanisms include upregulation of compensatory inhibitory checkpoints such as T-cell immunoglobulin and mucin-domain containing-3 (TIM-3), lymphocyte-activation gene-3 (LAG-3) and T-cell immunoreceptor with Ig and ITIM domains (TIGIT) (93–95), disruption of antigen-presentation machinery through loss of MHC-I or β2-microglobulin (96, 97), and expansion of immunosuppressive cellular subsets, including tumor-associated macrophages (TAMs), myeloid-derived suppressor cells (MDSCs) and regulatory T cells (Tregs) (98).

*Immunosuppressive myeloid cells* in the tumor microenvironment are particularly prominent mediators of resistance. Cells such as tumor-associated macrophages (TAMs) and myeloid-derived suppressor cells (MDSCs) markedly constrain the efficacy of immunotherapy (99, 100), particularly in metastatic castration-resistant prostate cancer (mCRPC) (101, 102). TAMs, often skewed toward an M2-like phenotype, promote tumor progression by enhancing angiogenesis, tissue remodeling and suppression of cytotoxic T-cell responses, whereas MDSCs comprise heterogeneous immature myeloid populations – monocytic and granulocytic – that inhibit antitumor immunity through arginase-1, iNOS, reactive oxygen species and immunosuppressive cytokines (103). Across malignancies, including mCRPC, higher TAM/MDSC abundance correlates with resistance to PD-1/PD-L1 therapy (102). Phenotypic and functional heterogeneity complicates therapeutic targeting of these myeloid compartments (104). Recent syntheses highlight TAM/MDSC-directed and myeloid checkpoint/trafficking axes, underscoring the complexity imposed by intra-/inter-subset heterogeneity (105, 106).

*Tumor-associated platelets*. An additional and mechanistically distinct pathway of immune escape involves tumor-educated platelets, also referred to as tumor-associated platelets (TAP). Sustained antigenic stimulation reprograms platelets toward a pro-tumorigenic phenotype, characterized by heightened expression of adhesion molecules and secretion of bioactive mediators that facilitate tumor progression (107, 108). Upon tumor cell intravasation, TAP rapidly form heteroaggregates with circulating cancer cells, shielding them from cytotoxic lymphocytes while simultaneously providing a reservoir of adhesive proteins that promote vascular arrest and metastatic colonization (109–111). This platelet-mediated cloaking represents a formidable barrier to durable immunotherapeutic efficacy (112, 113). Collectively, these mechanisms generate a profoundly inhibitory tumor microenvironment that undermines T-cell effector function despite checkpoint inhibition (92).

### 3.4 Additional constraints: 2025 update

#### 3.4.1 Concomitant medications and the microbiome

Emerging data up to 2025 clarify how several modifiable and context-dependent factors influence the efficacy, safety, and delivery of PD-1/PD-L1 blockade. Modifiable determinants of outcome include concomitant medications and the intestinal microbiota. The most consistent associations link proton-pump inhibitors and antibiotics with poorer survival on ICI, whereas the effects of glucocorticoids and opioids vary by indication, dose and timing of administration, supporting medication review and targeted microbiome-centric interventions in susceptible cohorts (114, 115).

#### 3.4.2 Infectious risk and HBV reactivation

Among individuals with chronic hepatitis B virus (HBV) infection, particularly those with hepatocellular carcinoma, HBV reactivation has been reported on ICI therapy; contemporary sources emphasize systematic screening, risk stratification and antiviral prophylaxis for carriers, even at low viraemia. Recent reviews and guidance advocate universal screening before systemic therapy and prophylaxis where indicated (116–118).

#### 3.4.3 Late immune-related adverse events

With longer treatment exposure, late irAEs increasingly manifest after at least 1–2 years of ICI, including in patients without early toxicities. Multicenter and lung cancer–specific cohorts suggest that roughly half of patients treated for more than 2 years may develop new late irAEs. This pattern necessitates prolonged surveillance, structured diagnostic pathways and individualized decisions on whether to continue beyond a fixed two-year horizon, with no universal stopping rule across tumor types (119–121).

#### 3.4.4 Fc engineering and format-dependent toxicity

On the molecule-engineering side Fc-silent formats and Fc re-engineering can reshape efficacy/toxicity profiles relative to classical IgG4, particularly for anti-PD-1/anti-TIGIT combinations, and support format selection tailored to the immune objective (122–124).

#### 3.4.5 Evolving biomarkers and digital pathology

Meanwhile, the biomarker landscape is shifting. Mature tertiary lymphoid structures and spatially resolved immune topologies provide additive prognostic/predictive value and, in some settings, outperform PD-L1 scoring; digital pathology/AI models trained on routine H&E can perform comparably to or better than PD-L1 and improve stratification when combined, including in lung cancer (125–127).

*Age and immunosenescence* prove more nuanced than expected: despite differing immune phenotypes and potential toxicity profiles in older adults, clinical outcomes are often comparable to those in younger cohorts, supporting geriatric stratification when deciding treatment duration and combinations (128–130). Together, these 2025 updates refine rather than replace the three core limitations described above and illustrate how evolving evidence continues to reshape the efficacy, safety, and implementation profile of PD-1/PD-L1 blockade.

## 4 Within the classical antibody approach: gains and gaps – why emerging modalities remain indispensable

### 4.1 Combination regimens with standard PD-1/PD-L1 antibodies

Within conventional PD-1/PD-L1 monoclonal antibodies, one intuitive strategy to overcome the limitations outlined above has been to embed them in combination regimens rather than to change the antibody format itself. n this section, we focus on clinical combinations of standard PD-1/PD-L1 IgG4 antibodies with other agents (for example, CTLA-4 blockade, chemotherapy, targeted therapies and myeloid modulators) and summarize where they partially mitigate pharmacokinetic, toxicity or resistance constraints and where important gaps remain. Some limitations can be addressed within antibody-based combination regimens (see **Supplementary Table S5** for an at-a-glance summary of late-phase combinations, key endpoints, and safety notes). Rational combinations address non-overlapping checkpoint biology and can counter selected intrinsic or adaptive resistance, albeit with toxicity trade-offs. In melanoma, anti-CTLA-4 plus anti-PD-1 delivers greater and more durable benefit than anti-PD-1 alone at 10 years, aligning with the division of labor between priming and effector phases (10).

### 4.2 Dual checkpoint blockade and chemo-immunotherapy: context-dependent gains

In routine practice, intensifying immune activation with dual checkpoint blockade (e.g., CTLA-4 plus PD-1) increases the rates and grades of immune-related adverse events and does not consistently outperform PD-1 monotherapy for overall survival across NSCLC settings; benefits remain program- and subgroup-specific (131). In gastrointestinal oncology, a 2025 systematic review reported that pembrolizumab monotherapy reduces adverse events yet does not improve overall survival, whereas pembrolizumab plus chemotherapy improves overall survival without a significant increase in overall toxicity versus chemotherapy alone (132). Taken together, these data support a context-dependent risk-benefit calculus: dual-ICI regimens often elevate immunotoxicity and warrant careful patient selection, while ICI–chemotherapy combinations can deliver survival gains with acceptable incremental toxicity when dosing, timing, and comorbidity are considered (see **Supplementary Table S5** for trial-level outcomes and irAE notes).

### 4.3 Pharmacokinetic constraints and exposure control within the classical antibody class

Pharmacology imposes additional design limits. Long antibody half-lives and sustained PD-1 receptor occupancy mean that discontinuation cannot rapidly abort immune-mediated toxicities; contemporary PK/PD analyses document prolonged PD-1 occupancy even at reduced or infrequent dosing, strengthening the rationale for formats with tighter exposure control (133). These pharmacokinetic constraints highlight intrinsic limits of IgG-based PD-1/PD-L1 antibodies and motivate exploration of compact scaffolds and rationally designed small-molecule PD-1/PD-L1 inhibitors to mitigate size-limited diffusion (134), alongside nanomedicine-based localization to confine checkpoint effects to the tumor microenvironment (135).

### 4.4 Hybrid strategies and microenvironment-aware approaches

Selective hybrid or adjacent strategies illustrate how pharmacology and biology can be aligned within and at the margins of the antibody class. Early clinical proof-of-concept indicates that adding the JAK1/2 inhibitor ruxolitinib can re-sensitize classical Hodgkin lymphoma to PD-1 blockade after prior CPI failure, reinforcing the role of JAK-STAT tuning in overcoming resistance (136). The adenosine pathway provides another lever: PD-L1 blockade combined with A2A antagonism (ciforadenant) has shown feasibility and preliminary activity with biomarker ties (137). Preclinical microenvironment-aware delivery platforms, including platelet-targeted nanoparticles (112) that enhance anti-PD-1 activity, are summarized in **Supplementary Table S6**.

Overall, these combination strategies show that standard PD-1/PD-L1 antibodies can be leveraged more effectively in specific contexts, but they do not fully resolve the three core limitations of pharmacokinetics and penetration, immune toxicity or adaptive and microenvironmental resistance. This motivates the subsequent section, which systematically examines within-class engineering of PD-1/PD-L1 antibodies (Fc modification, bispecifics and conjugate-like designs) and contrasts their mechanistic advantages and trade-offs.

## 5 Alternative antibody formats for PD-1/PD-L1

### 5.1 Fc modification and isotype engineering

Fc-engineered monospecific PD-1 antibodies, such as FcγR-attenuated IgG4 formats exemplified by tislelizumab, aim to reduce FcγR-mediated clearance of PD-1^+^ effector T cells and mitigate Fc-driven toxicities while preserving checkpoint blockade. Their main advantages and remaining gaps relative to conventional IgG4 PD-1/PD-L1 mAbs together with those of checkpoint-targeting bispecific and conjugate or tumor-restricted PD-1/PD-L1 formats discussed below, are summarized in **Table 1**. Studies of FcγR interactions along the PD-1/PD-L1 axis have shown that Fc-engaging anti-PD-1 antibodies can paradoxically limit antitumor activity, in contrast to Fc-competent anti-PD-L1 or anti-CTLA-4 mAbs where depletion of suppressive myeloid or regulatory populations may be beneficial (138).

**Table 1:** Comparative summary of Fc-modified monospecific ABs, checkpoint-targeting bsABs, and conjugate or tumor-restricted PD-1/PD-L1 ABs: mechanistic advantages and practical trade-offs.

Strategy	Example	Main limitation addressed	Mechanistic advantages vs standard PD-1/PD-L1 mAbs	Key trade-offs/gaps
Fc-engineered PD-1 mAbs	Tislelizumab, FcγR-attenuated IgG4 anti–PD-1 (143)	Immune toxicity and FcγR-mediated clearance of PD-1^+^ effector T cells; little effect on penetration	Preserve a monospecific format while reducing FcγR engagement and antibody-dependent phagocytosis of PD-1^+^ T cells; may modestly lower some irAEs and Fc-driven resistance	Benefits typically incremental; size, PK and tissue penetration similar to conventional PD-1 IgG4; each Fc variant adds CMC and regulatory complexity; sanctuary-site and microenvironmental barriers remain
Checkpoint bsAbs	Cadonilimab, Fc-null PD-1/CTLA-4 bsAb (144); conceptually similar late-stage PD-L1-anchored multi-specifics (for example, PD-L1×costimulator formats such as 4-1BB-containing tri- or tetraspecifics)	Adaptive/microenvironmental resistance via redundant PD-1 and CTLA-4 signaling; partial toxicity reshaping	Co-blockade of PD-1 and CTLA-4 in a single molecule with preferential retention in PD-1/CTLA-4-high TME and limited Fc effector function; can deepen responses beyond PD-1 monotherapy in selected settings	irAEs more frequent than with PD-1 alone; narrow therapeutic window and complex dosing/selection; PK and penetration still IgG-like, so systemic exposure and organ barriers not fundamentally overcome
Conjugate and conditionally active formats (immunocytokines, masked or tumor-restricted antibodies)	PD1-IL2v, PD-1–targeted IL-2 variant immunocytokine (145) Pacmilimab (CX-072), protease-activated masked PD-L1 antibody (146)	Adaptive resistance in “cold” or exhausted tumors; systemic IL-2 toxicity; low local immunostimulatory concentration; Immune toxicity and organ safety by lowering activity in healthy tissues; partial PK/penetration leverage via higher tolerated tumor-directed exposure	Couple PD-1 blockade with spatially restricted IL-2Rβγ agonism on PD-1^+^ stem-like CD8^+^ T cells, potentially re-inflaming poorly infiltrated tumors and reshaping T-cell states more deeply than PD-1 alone; Masked PD-L1-binding domain designed to be unmasked by tumor-associated proteases, aiming to concentrate functional antibody activity in the TME and allow higher local occupancy with fewer systemic irAEs than unmasked PD-1/PD-L1 mAbs	Therapeutic window and optimal schedule uncertain; cytokine-related toxicities remain possible; manufacturing and analytics complex; evidence mostly preclinical or early phase, so long-term benefit vs standard PD-1/PD-L1 mAbs unclear; Degree and consistency of tumor-selective activation still being defined; added molecular complexity and potential immunogenicity; distribution remains IgG-like, so deep-tissue penetration is still constrained; optimal combinations and long-term outcomes vs standard PD-1/PD-L1 blockade not established

#### 5.1.1 Clinically advanced examples

Tislelizumab (BGB-A317) is a humanized IgG4 anti-PD-1 antibody in which lower-hinge Fc residues were substituted to minimize FcγR binding on macrophages and thereby reduce antibody-dependent phagocytosis of PD-1^+^ T cells, while retaining high-affinity PD-1 engagement and durable receptor occupancy (139).

Penpulimab provides a complementary example of an Fc-engineered IgG1 anti-PD-1 antibody, in which the Fc region is rendered FcγR/C1q-null; early clinical data suggest effective checkpoint blockade with a low incidence of immune-related adverse events and favorable physicochemical stability compared with conventional IgG4 backbones (140).

Preclinical and mechanistic work with Fc-null anti-PD-1 mAbs further supports this trajectory: Fc-null frameworks prevent FcγR-mediated resistance and deliver more consistent T-cell activation across “hot” and “cold” tumor models than Fc-competent IgG4 S228P backbones, and IgG4-mediated Fc–Fc interactions can dampen ADCC and ADCP by partner antibodies, whereas Fc-null IgG1 and Fc-null IgG4 variants such as penpulimab and tislelizumab avoid these inhibitory Fc–Fc effects; together, these data provide a rationale for Fc-effector–silent frameworks in PD-1 blockade (141, 142). In functional terms, Fc modified PD-1 antibodies mainly aim to reduce Fc driven toxicity and loss of PD-1 positive effector T cells, while leaving pharmacokinetics and tissue penetration largely unchanged.

### 5.2 Bispecific and multispecific antibodies targeting the PD-1/PD-L1 axis

Bispecific antibodies (bsAbs) that bind PD-1/PD-L1 together with a second checkpoint or a costimulatory receptor are designed to co-localize checkpoint blockade and conditional engagement of immune cells within the tumor microenvironment, potentially increasing therapeutic efficiency while limiting off-tumor activity. Similarly to anti-PD-1 mAbs (138, 141), bsAbs frequently employ Fc effector-null (FcγR/C1q-silent) formats to minimize Fc-mediated toxicities without compromising target engagement (144).

#### 5.2.1 Representative clinical programs

A symmetric tetravalent PD-1×CTLA-4 antibody, cadonilimab (AK104), exploits trans-binding on dual-positive T cells, with tetravalency and an Fc-null design intended to intensify local checkpoint blockade while attenuating Fc-driven effector functions; mechanistically, it is engineered to enhance PD-1 blockade and interfere with CTLA-4 interactions with CD80/CD86, although definitive late-phase, head-to-head superiority over separate anti-PD-1 plus anti-CTLA-4 mAbs has not been established (144). Additional binding logics illustrate the modularity of bsAbs: PD-L1×4-1BB constructs such as acasunlimab (GEN1046) deliver PD-L1-anchored, 4-1BB agonism intended to confine the costimulatory activity of 4-1BB to PD-L1-positive cells and thereby mitigate systemic toxicity; first-in-human data reported manageable safety and early antitumor activity (147, 148). Similarly, the DART-format bsAb tebotelimab (MGD013), which targets PD-1 and LAG-3, achieved concurrent receptor occupancy and demonstrated antitumor activity across solid and hematologic malignancies in a phase-1 study; however, no head-to-head evidence has shown superiority over monotherapies, and claims of enhanced rescue of exhausted T-cell function should be interpreted within the limits of early-phase datasets (149). Co-blockade of LAG-3 with PD-1 outperforms PD-1 monotherapy with a more favorable irAE profile; PD-1×LAG-3 bispecifics are mechanistically concordant with this biology (150). By contrast, adding TIGIT or TIM-3 remains biologically plausible yet performance in late-phase clinical trials has been inconsistent, underscoring the need for biomarker-guided selection, for example, integrity of the CD226 (DNAM-1) axis (151, 152).

#### 5.2.2 Late-phase signals and higher-order multispecifics

Clinically, cadonilimab has completed early-phase evaluation with manageable safety and antitumor activity across solid tumors (144). A randomized phase-3 trial subsequently showed that adding cadonilimab to platinum-based chemotherapy with or without bevacizumab in first-line persistent/recurrent/metastatic cervical cancer significantly improved progression-free and overall survival with a manageable safety profile (153, 154). Late-phase development continues across first-line and consolidation settings, including combinations with chemotherapy and targeted agents (155, 156). KN046 (PD-L1×CTLA-4) demonstrated clinically meaningful activity and tolerability with chemotherapy in metastatic non-small-cell lung cancer (157) and favorable outcomes in triple-negative breast cancer, particularly in PD-L1-positive disease (158). Tebotelimab progressed through phase-1 testing, eliciting objective responses in PD-1-refractory malignancies and activity in LAG-3-positive lymphomas (149). In parallel, acasunlimab moved into phase 2 investigation following proof-of-concept data consistent with PD-L1-anchored, context-dependent 4-1BB agonism and durable disease control in heavily pretreated patients (147, 148). This design logic extends to higher-order multispecifics, including trispecific and tetraspecific antibodies (106). Particularly, the octavalent tetra-specific CD19xCD3x4-1BBxPD-L1 antibody emfizatamab is currently developed to target CD19+ lymphoma and leukemia cells by the engagement and activation of T cells via their CD3 and 4-1BB receptors, while also preventing tumor T cell inhibition by blocking PD-L1 (NCT04606433) (159, 160).

#### 5.2.3 Advantages and limitations

Collectively, checkpoint-targeting bispecific and multispecific antibodies can provide (i) enhanced selectivity via requirement for target co-expression (144); (ii) mitigation of systemic adverse events through Fc- null or conditional-activation formats (147, 148); (iii) improved intratumoral pharmacodynamics attributable to high avidity and co-localized checkpoint interference (144); and (iv) the capacity to address resistance to PD-1 monotherapy by concomitant blockade of compensatory inhibitory receptors or incorporation of costimulatory pathways (149, 157). Limitations include the need for robust biomarkers to identify relevant co-expression patterns (149), optimization of dose/exposure to balance efficacy with immune-related toxicity (147), and confirmatory evidence from late-phase randomized trials to demonstrate superiority over established dual-antibody regimens (157). With phase-3 reporting in 2024–2025, including COMPASSION-15 in first-line HER2-negative gastric/gastroesophageal junction adenocarcinoma (154) and COMPASSION-16 in first-line persistent/recurrent/metastatic cervical cancer (153), checkpoint-targeting bispecifics are beginning to define late-phase benchmarks that will determine their clinical positioning across tumor types. Conjugate or conditionally active formats may be built on mono- or multispecific backbones (145, 146, 161, 162). Checkpoint bispecific and multispecific antibodies are thus directed mainly at adaptive and microenvironmental resistance rather than basic exposure, by co blocking compensatory checkpoints or adding context dependent costimulation in the tumor microenvironment.

### 5.3 Conjugate, conditionally active, and gene-encoded PD-1/PD-L1 formats

#### 5.3.1 Dual-mechanism protein conjugates

Beyond checkpoint–checkpoint and checkpoint-costimulator pairs, dual-mechanism molecules such as PD1-IL2v immunocytokines couple PD-1 binding to IL-2Rβγ-biased signaling in cis, deepening T-cell reprogramming while attempting to limit systemic IL-2 toxicity (145, 161). Preclinical, translational and first-in-human studies with PD1-IL2v show enhanced expansion of stem-like CD8 T cells and suggest potential utility after checkpoint inhibitor failure (145, 163).

#### 5.3.2 Angiogenesis-targeting and masked designs

In a different example of dual-mechanism therapeutics, a bispecific antibody can combine PD-1 binding with another functional unit targeting non-immune processes, such as tumor angiogenesis. Vascular normalization coupled to PD-1 blockade via the PD-1×VEGF-A bispecific ivonescimab improved progression-free survival in first-line PD-L1-positive NSCLC, illustrating microenvironmental reprogramming as a lever for efficacy (164) (see **Supplementary Table S6**). However, as of 2025, the therapy is not approved in the United States or European Union (165–167).

Tumor-activated “prodrug” antibodies, such as the protease-activated anti-PD-L1 Probody pacmilimab (CX-072), further exemplify conjugate or masked designs that aim to retain systemic exposure while reducing off-tumor toxicity by unmasking the antigen-binding site preferentially within the tumor microenvironment (146). Taken together, immunocytokines, angiogenesis-targeting bispecifics and protease-activated antibodies form a conjugate-like subclass in which PD-1/PD-L1 recognition is physically linked to a second functional module, with the shared goal of intensifying intratumoral activity while attempting to preserve or improve the systemic therapeutic window.

#### 5.3.3 Gene- and nucleic-acid-encoded fragments and nanobodies

Beyond these protein-level conjugates, gene-encoded or locally delivered antibody fragments are being clinically explored to concentrate checkpoint blockade within the tumor while limiting systemic toxicity. The chimeric vaccinia virus CF33-hNIS-antiPDL1 allows secretion of an anti-PD-L1 scFv and demonstrates functional PD-L1 blockade with T-cell activation in malignant ascites from patients with gastric cancer (168). The efficacy of this approach was further supported *in vivo* in an orthotopic pancreatic ductal adenocarcinoma model in 2025 (169). The oncolytic HSV-1 vector T3011 co-expresses IL-12 and an anti-PD-1 antibody fragment; first in-human data show acceptable tolerability and evidence of biological activity across solid tumors, supporting the feasibility of *in situ* production of checkpoint-blocking payloads (170, 171). In a gene-encoded format, systemic AAV8 delivery of an anti-PD-1 nanobody (Nb11) under a CAG promoter maintained serum levels in the μg/mL range and prevented tumor formation in ~30% of mice after MC38 challenge, with significant survival benefit and low toxicity (172). Nb11 thus remains a preclinical exemplar of long-acting, low-dose immunomodulation via continuous expression (173, 174).

Complementary strategies now use mRNA or circular RNA constructs to encode anti-PD-L1 nanobodies for local delivery. In colorectal cancer models, lipid nanoparticle delivery of anti-PD-L1 nanobody mRNA and circRNA vectors co-encoding IL-15 and an anti-PD-L1 nanobody have produced robust antitumor responses and reshaped myeloid and T-cell infiltrates while maintaining a predominantly local pharmacologic footprint (175, 176). Taken together, conjugate, conditionally active and gene-encoded formats aim to widen the therapeutic window and overcome resistant tumor niches by concentrating activity in the tumor bed and in some cases reshaping a poorly inflamed microenvironment.

### 5.4 Compact antibody formats: nanobodies and other reduced-size scaffolds

#### 5.4.1 Diffusion and epitope access

Alternative antibody formats comprising single-chain variable fragments (scFv), antigen-binding fragments (Fab) and single-domain antibodies (sdAb, VHH, often called ‘nanobodies’) are engineered to address limitations of full-length IgG in checkpoint blockade (162). VHH denotes the variable domain of camelid heavy-chain antibodies; their compact architecture and modularity enable variable PD-1/PD-L1 formatting and control of pharmacokinetics via Fc-fusion, albumin-binding modules, or PEGylation. For clinically relevant half-life extension, albumin binding is often preferred (177). By virtue of their lower hydrodynamic radius, sdAb/scFv/Fab can diffuse more effectively through dense extracellular matrix and achieve more homogeneous intratumoral distribution than conventional IgGs, although rapid renal clearance still necessitates half-life extension for systemic use (177–179). For example, in MC38 colorectal carcinoma models, an anti-PD-L1 VHH penetrated deeper into tumor parenchyma and achieved greater therapeutic efficacy than a full-length IgG (180), consistent with the size-enabled diffusion advantage of sdAb/scFv.

**Figure 2:**
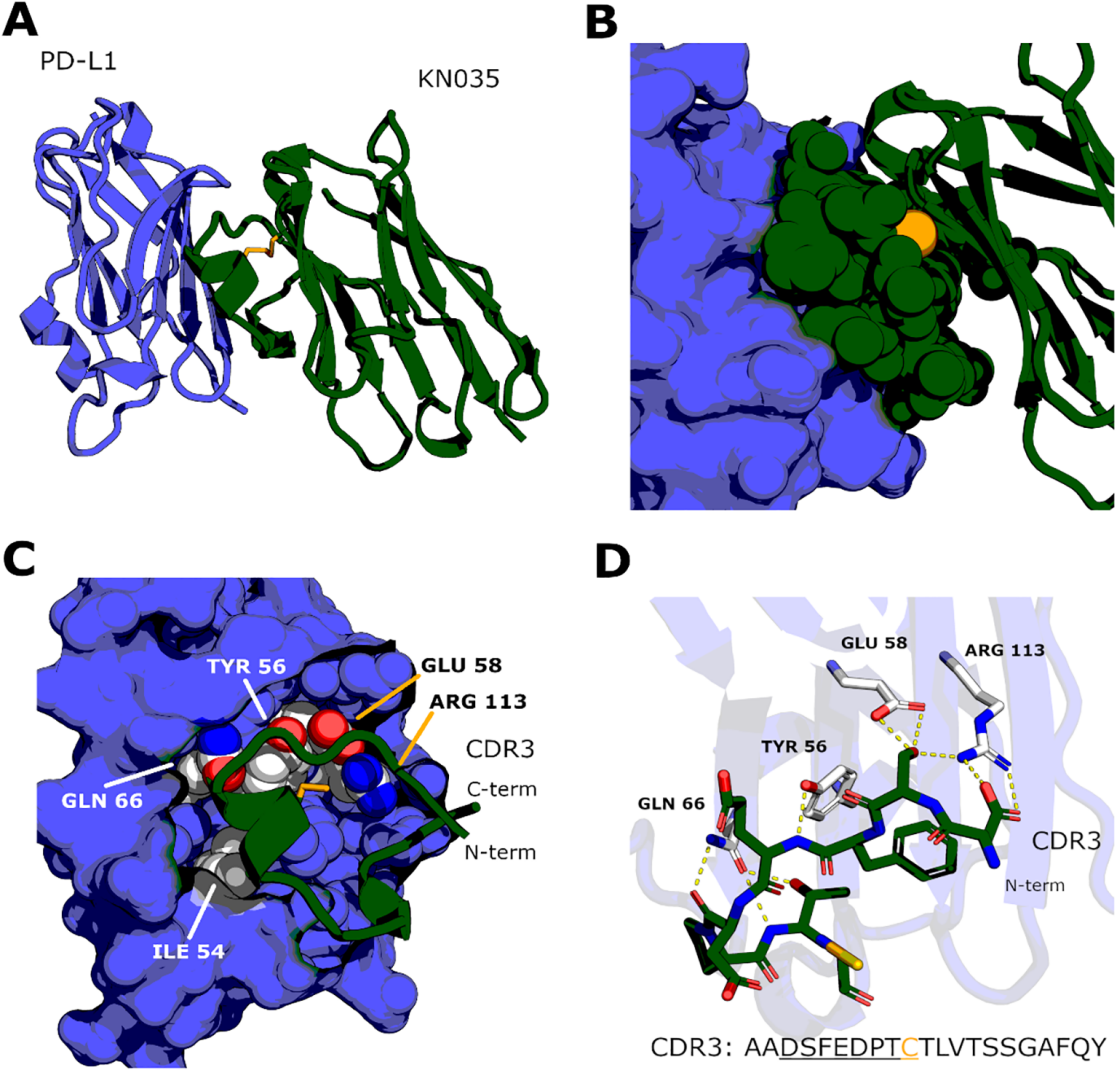
Structural basis of PD-L1 recognition by the KN035 nanobody (envafolimab). **(A)** Overall architecture of the PD-L1/KN035 complex (PDB:5JDS) shown in ribbon representation. The nanobody contacts the front β-sheet of PD-L1 comprised of CC’FG strands; the epitope overlaps with PD-1 binding site. **(B)** The epitope on PD-L1 (shown as blue surface) is almost flat, and nanobody forms the binding interface almost exclusively via a long (18 a.a.) CDR3 (shown as green spheres) stabilized by a disulfide bond Cys38–Cys111.3. **(C)** CDR3 (shown as ribbon) forms multiple contacts with five “hotspot residues” on the epitope: I54, Y56, E58, Q66, R113 (shown as spheres); mutation of each of these residues to alanine decreases affinity by at least 40-fold. **(D)** The N-terminal half of CDR3 (residues DSFEDPTC) forms multiple hydrogen bonds (dashed lines) with four hotspot residues. These interactions partly explain 1000-fold higher affinity for PD-L1/KN035 compared to PD-L1/PD1 complex (181).

Selection of sdAb and scFv may also enable recognition of cryptic epitopes on PD-1/PD-L1 that are relatively inaccessible to full-length antibodies, potentially enhancing functional blockade with reduced steric hindrance (162). For KN035 (envafolimab), structural analysis supports such engagement of a non-standard cryptic epitope (181). This likely reflects a favorable structural complementarity: a flat epitope on the front β-sheet of PD-L1 (formed by CC′FG strands) is bound primarily by an unusually long CDR3 of 18 amino acid residues in KN035, which is stabilized by an additional Cys38-Cys111.3 disulfide bond. Folded CDR3 with two short helical fragments in KN035 forms a virtually flat contact surface with a dense hydrogen-bonding network formed by five hot-spot residues (I54, Y56, E58, Q66, R113), that effectively overlaps with the PD-1 binding site on PD-L1 (**Figures 2A–D**).

#### 5.4.2 Envafolimab as clinical exemplar

Advances in nanobody engineering including fully human VH-only libraries, CDR grafting, and framework resurfacing have reduced immunogenicity and improved developability in VHH-based formats intended for chronic oncology dosing (182–184). The most clinically advanced PD-1/PD-L1 nanobody, envafolimab, a humanized anti-PD-L1 sdAb fused to human IgG1 Fc for subcutaneous administration, has received regulatory approval in China for previously treated MSI-H/dMMR solid tumors (174). In 2024, model-informed pharmacokinetic (PK)/pharmacodynamic (PD) and exposure–response analyses supported fixed or alternative dosing and underscore the practicality of sdAb-Fc formats for outpatient immunotherapy (185). Additional 2025 clinical reports indicate ongoing program expansion (186). Envafolimab is currently the only PD-1/PD-L1-directed nanobody-based therapeutic to have reached clinical use, and a clinical snapshot of envafolimab programs is summarized in **Table 2**. Compact nanobody-based formats are therefore positioned primarily to ease subcutaneous dosing and to improve distribution in solid tumors, while their impact on resistance biology is still being defined.

**Table 2:** Clinical trial snapshot – envafolimab (KN035, sdAb−Fc).

ClinicalTrials.gov ID	Phase	Status	Indication	Link
NCT02827968	I	Completed	Multiple solid tumors	(187)
NCT03248843	I/II	Completed	Multiple solid tumors (Japanese cohort)	(188)
NCT03667170*	II	Completed	MSI−H/dMMR solid tumors	(173)
NCT04480502**	II	Terminated (ENVASARC)	Soft tissue sarcomas	(189)
NCT06123754	III	Active, not recruiting	Non–small−cell lung cancer (NSCLC)	(190)

*Trial with positive primary outcomes/regulatory−enabling results; ** The developer announced discontinuation due to failure to meet the primary endpoint (ORR) in July 2024.

Alternative PD-1/PD-L1 formats expand the design space compared with full-length IgG by improving epitope access, tumor penetration and dosing flexibility, but they introduce non-trivial developability and delivery constraints. For PD-1/PD-L1 specifically, late-phase randomized evidence remains limited compared with full-length mAbs, making definitive positioning contingent on outcomes from ongoing and upcoming studies (162). In addition, manufacturability, stability, delivery logistics and infrastructure requirements are likely to be limiting factors for broad deployment even when early efficacy signals are encouraging.

## 6 Small−molecule inhibitors of PD−1/PD−L1: compact modalities with delivery advantages

Stepping outside antibody formats, subcutaneous formulations such as envafolimab streamline clinic operations and align with patient preferences (185), yet they do not overcome intratumoral diffusion barriers or systemic resistance (191), which sustains the case for compact chemical entities able to penetrate dense tumor stroma and be given orally or locally (192). Compared with IgG formats, compact ligands offer distinct delivery physics and controllability (**Supplementary Table S7**). Overall, small-molecule PD-1/PD-L1 inhibitors are therefore positioned to address penetration and distribution constraints of antibody formats and to enable flexible oral or local dosing while maintaining checkpoint blockade at the target site (193, 194).

### 6.1 Mechanistic classes and structural exemplars

**Figure 3:**
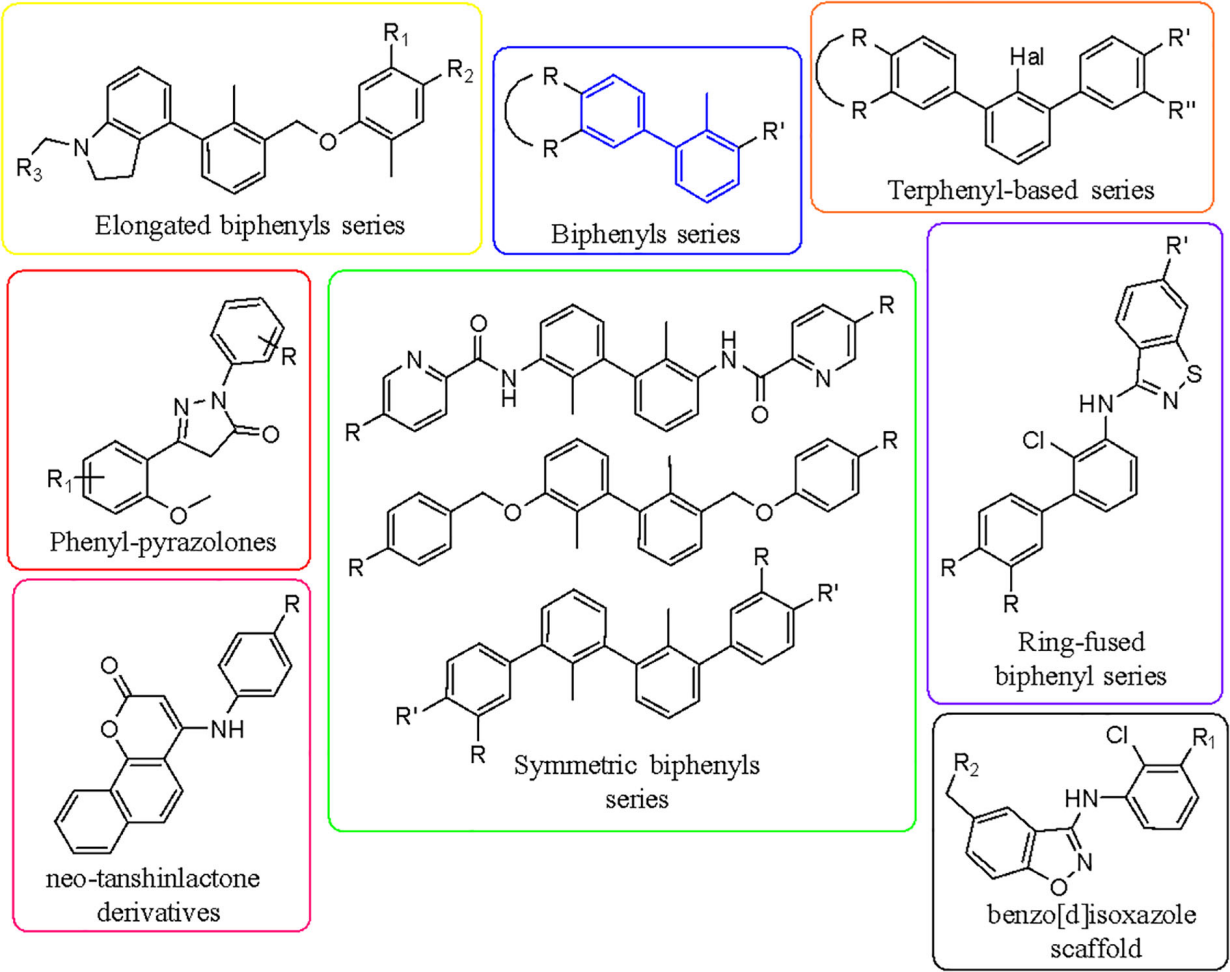
Main classes of actual PD-L1 inhibitors starting from BMS-like concept. Schematic overview of PD-L1 inhibitor classes anchored by the BMS-like small-molecule concept (PD-L1 dimerization and interface occlusion), with branches to macrocycles/peptides, imaging ligands, and other emerging chemotypes. The diagram groups modalities by mechanism and maturation stage, highlighting how BMS-like scaffolds served as the starting point for subsequent PD-L1-targeting designs.

**Figure 4:**
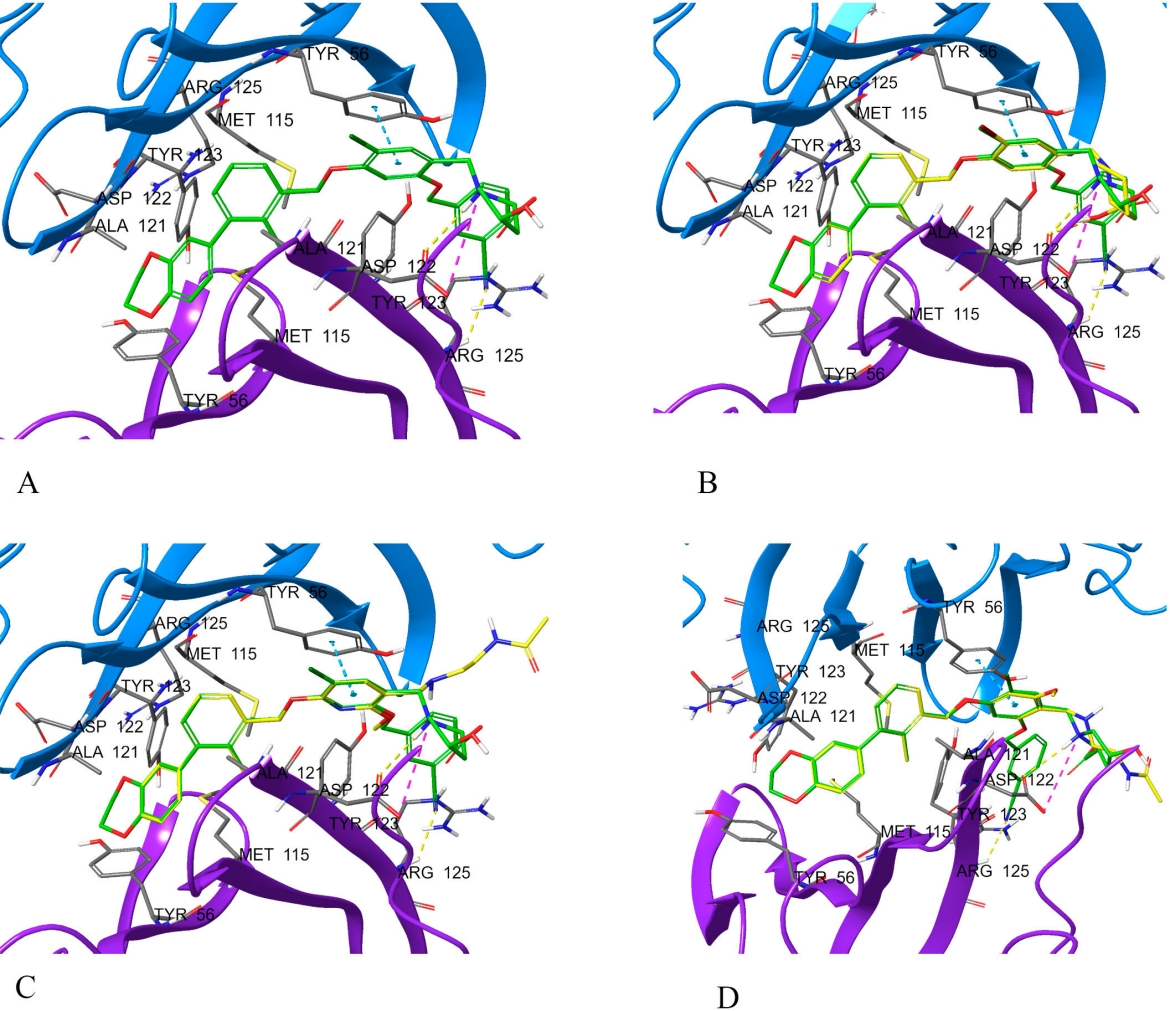
PD-L1 dimer complexed with: **(A)** BMS1166, **(B)** BMS8, **(C)** BMS202, **(D)** BMS37. Compounds are overlaid (BMS-1166 in green). Dashed lines: yellow, hydrogen bonds; azure, π-stacking; magenta, π-cation. Chains: A (violet), B (blue). Overlay of four BMS-series ligands at the PD-L1 homodimer interface, highlighting a shared pocket across chains A (violet) and B (blue) and interaction types (yellow hydrogen bonds, azure π-stacking, magenta π-cation), with BMS-1166 shown in green.

One of the first PD-1/PD-L1 inhibitors was the (2-methyl-3-biphenylyl)methanol scaffold (**Figure 3**), developed by Bristol-Myers Squibb (BMS). The most extensively validated small-molecule mechanism is to occupy a hydrophobic pocket on PD-L1 and induce PD-L1 dimerization, sterically shielding the PD-1 interaction surface and thereby disrupting PD-1/PD-L1 signaling; crystallography and NMR establish this mode for BMS exemplars, including BMS-202 (1) and BMS-8 (2), and related series (14, 195, 196). At the residue level, PD-L1 dimerizers occupy a hydrophobic tunnel formed by Tyr56, Met115, Ala121 and Tyr123, with polar linkers engaging Asp122 and Arg125, a ‘dimer-lock’ that sterically occludes PD-1 (**Figure 4**).

**Figure 5:**
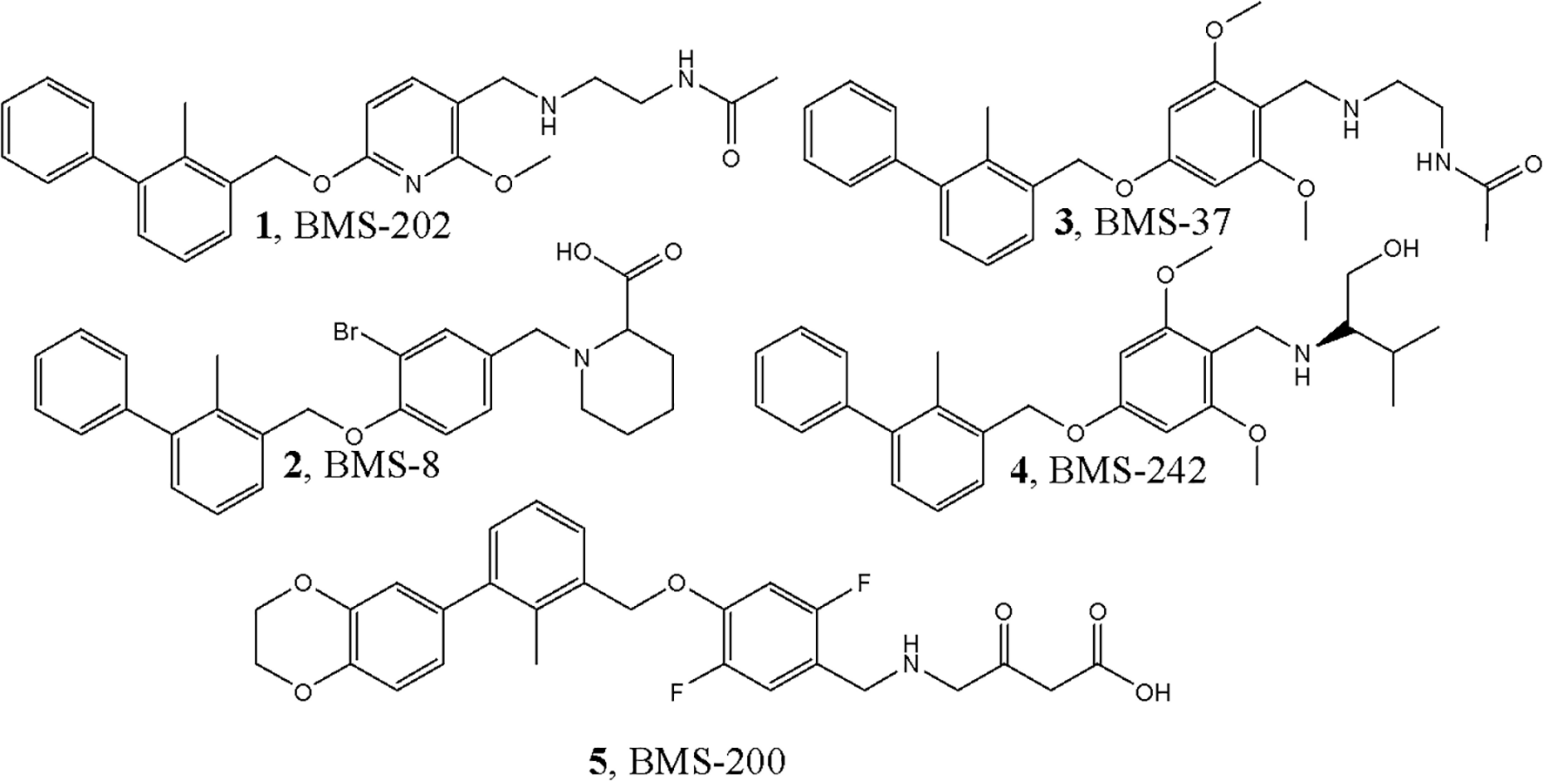
Representative “BMS-series” small-molecule inhibitors of the PD-1/PD-L1 interaction (BMS-202, BMS-8, BMS-37, BMS-242, BMS-200), illustrating the shared biphenyl chemotypes and substituent patterns characteristic of PD-L1-dimerizing scaffolds.

**Figure 6:**
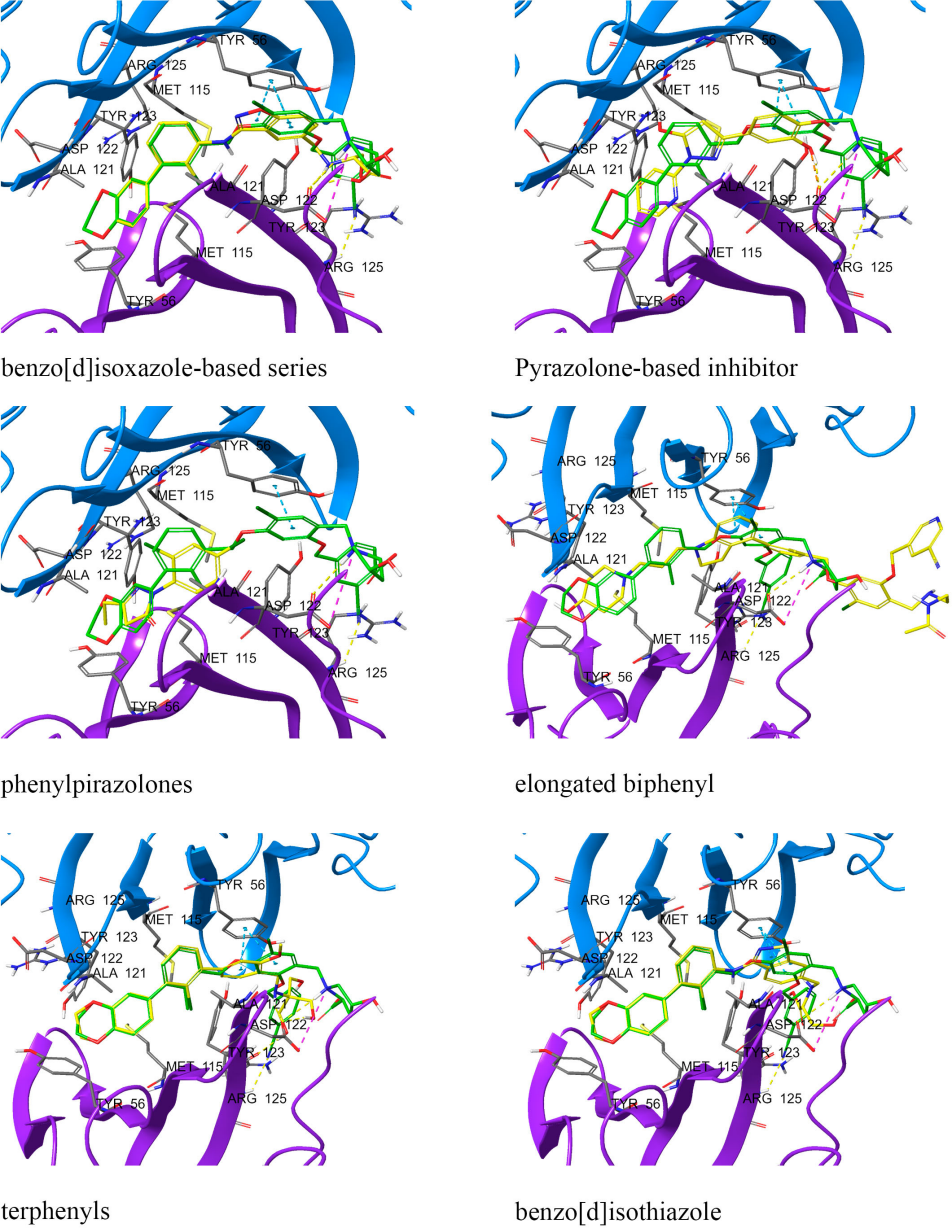
Lead compounds showed high binding affinity to PD-L1. Comparison of binding affinities for lead PD-L1–targeting compounds, where lower Kd/IC50 values indicate stronger binding. Several candidates cluster in the low-nanomolar range, highlighting the strongest PD-L1 engagement.

The “BMS series” (**Figure 5**) are archetypal biphenyl dimerizers of PD-L1, and recent asymmetric biphenyl designs preserve the dimerization paradigm while opening new vectors for ADME and safety optimization (14, 197). Representative PD-L1–ligand crystal complexes and associated activity metrics are summarized in **Table 3** (e.g., PDB 5NIU, 6R3K). Binding-affinity profiling highlights several lead PD-L1–targeting compounds clustering in the low-nanomolar range (**Figure 6**).

**Table 3:** Crystallographic complexes of PD-L1 with small-molecule ligands (BMS series): structure and activity.

№#	PDB ID	Resolution	Compound	Date	Activity	References
1	5J89	2.2	1, BMS-202	2016.04	18 nM	(14)
2	5J8O	2.3	2, BMS-8	2016.04	146 nM	(14)
3	5N2D	2.35	3, BMS-37	2017.06	6–100 nM	(202)
4	5N2F	1.70	5, BMS-200	2017.06	80 nM	(202)
5	5NIU	2.01	6, BMS-1001	2017.12	2.25 nM	(195)
6	6NM8	2.79	8, BMS small molecule 105	2019.02	53 nM	(203)
7	6R3K †	2.20	7, BMS-1166	2019.04	3.89 nM	(204)

† Previously deposited as 5NIX (same complex).

PDB ID – Protein Data Bank identifier for the human PD-L1–ligand complex (a second code in parentheses indicates an alternative deposition of the same structure); Resolution – X-ray resolution in ångströms (Å); Compound – ligand name and internal serial number from the original publication (for example, BMS-202, BMS-8); Date – month and year of structure deposition/publication; Activity – reported potency against the PD-1/PD-L1 axis (usually IC_50_, occasionally K_D or EC_50_, in nM). Because different assay formats are used (for example, HTRF, SPR, cell-based), activity values are approximate and not strictly cross-comparable.

Beyond biphenyls, multiple chemotypes have been reported, including pyrazolones with biophysical evidence of PD-1/PD-L1 disruption measured by microscale thermophoresis (MST) or Förster resonance energy transfer (FRET) (198), indanes as rigidified scaffolds with PD-1/PD-L1 activity (199), and symmetric phenyl-linked ligands with high PD-L1 affinity (200); contemporary reviews map the binding interactions and chemical space across these families (134, 201). Structure-guided rigidification, for example by moving from biphenyl to terphenyl scaffolds or by ring fusion, can strengthen pi-pi contacts with PD-L1 Tyr56 and open vectors for potency and pharmacokinetic optimization (134, 197). Two non-peptidic mechanistic classes dominate current efforts, namely PD-L1 dimerizers and steric blockers that occlude the PD-1 contact surface (198, 199).

Alternative small-molecule routes are also emerging. Certain ligands trigger PD-L1 internalization and degradation, potentially extending pharmacodynamic effects beyond plasma exposure, and cell-based studies show that ligand-induced dimerization can accelerate loss of PD-L1 from the membrane; structural analyses further rationalize how small molecules remodel PD-L1 surfaces to preclude PD-1 binding (205–208).

Relative to monoclonal antibodies, small molecules promise oral dosing, deeper penetration through fibrotic stroma and poorly perfused tumor regions, a short and controllable half-life for safety management (192), and scalable manufacturing with fine-grained medicinal-chemistry tuning (134). Disease-context data also suggest tumor-type-specific benefits, for example supportive biology for BMS-202 in glioblastoma models (20).

### 6.2 Clinical development and outstanding challenges

**Figure 7:**
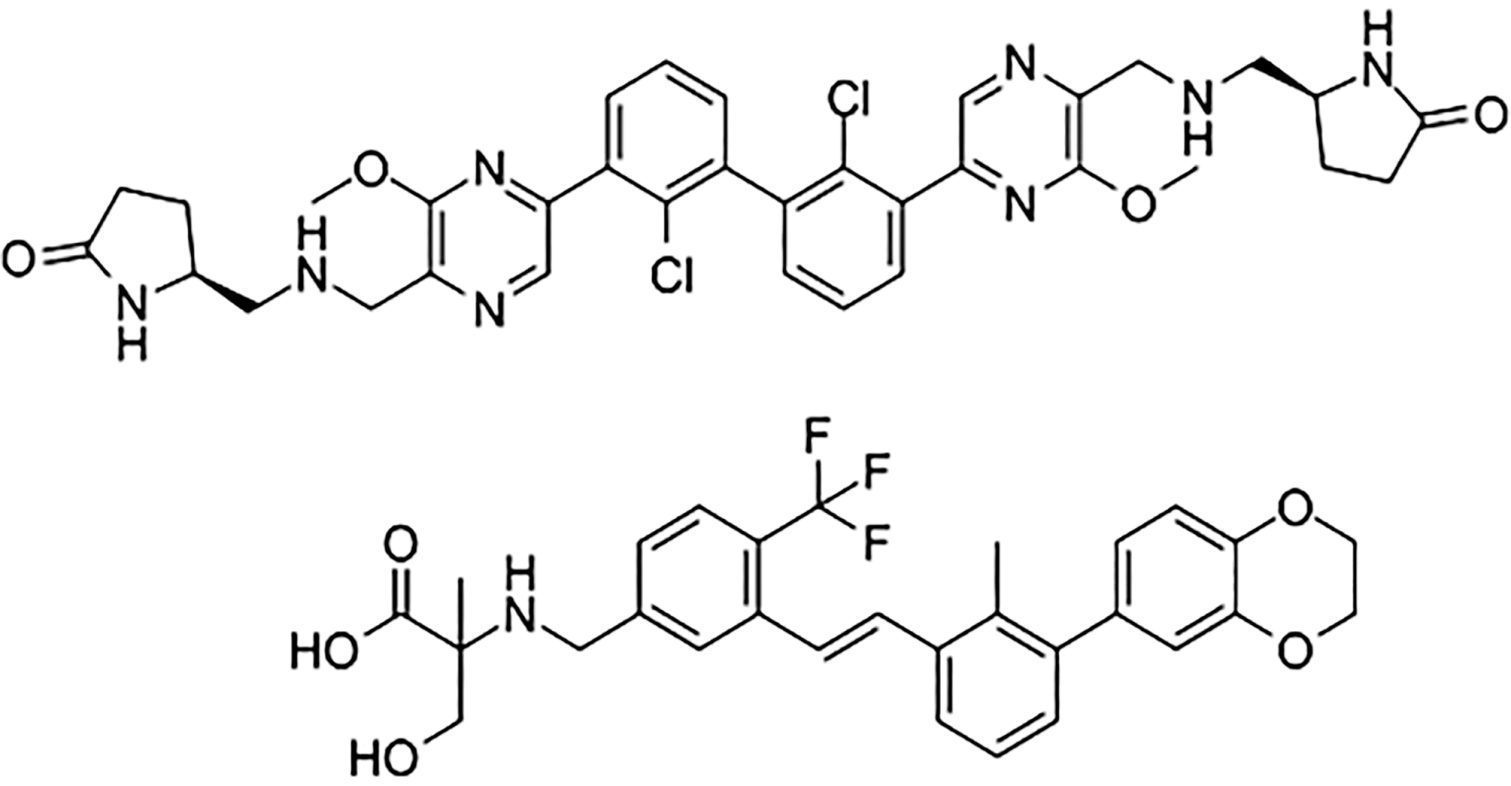
Chemical structures of two oral PD-L1 candidates in first-in-human testing: evixapodlin (GS-4224) and MAX-10181. Side-by-side chemical structures of evixapodlin and MAX-10181; clinical identifiers and status are provided in **Table 3**.

Clinically, no approved small-molecule PD-1/PD-L1inhibitors exist as of 2025, but several programs offer on-target pharmacodynamic evidence in humans. The PD-L1 ligand INCB086550 demonstrated target-engagement signatures and antitumor activity in preclinical and translational studies with early clinical readouts (209), whereas the oral PD-L1 inhibitor evixapodlin (GS-4224) reported PD-L1 engagement and expected immune pharmacodynamics in first-in-human testing; other oral agents such as MAX-10181 (see **Figure 7**) are progressing through phase-1 development (210, 211).

**Figure 8:**
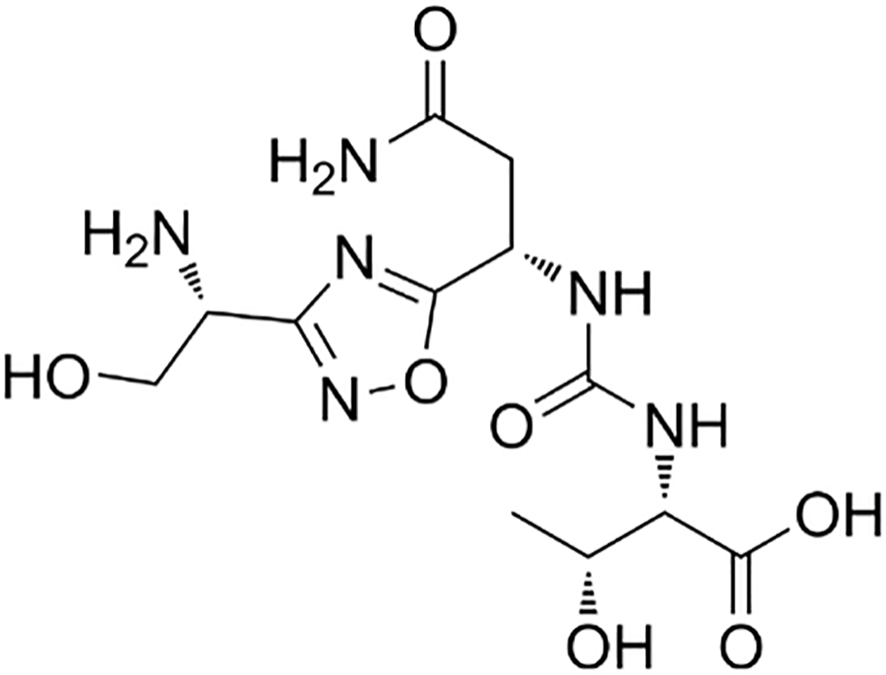
CA-170 PD-L1/VISTA dual inhibitor. Schematic of CA-170 as an oral small-molecule modulator positioned on the PD-1/PD-L1 and VISTA axes.

For combination strategies with antibody formats, small molecules can complement prolonged systemic control by bridging pharmacokinetic windows between infusions and diffusing into ‘cold’ regions that are less accessible to IgG, a rationale supported by preclinical synergy, for example an oral PD-L1 inhibitor MAX-10181 enhancing PD-1×CTLA-4 bispecific therapy in animal models, and by mechanistic data demonstrating distinct effects of dimerizers on PD-L1 membrane dynamics (159, 207). Notwithstanding these advances, the shallow and extended nature of the PD-1/PD-L1 interface and the need to stabilize PD-L1 dimers help explain why discovering potent, drug-like disruptors remains challenging. Disrupting this large protein-protein interface with drug-like molecules carries risks of insufficient potency, off-target cytotoxicity and discordance between biophysical readouts and T-cell functional rescue (196). Critiques of CA-170 (**Figure 8**) highlight a lack of direct PD-1/PD-L1 inhibition in independent systems (212) despite early clinical exploration, for example NCT02812875 (213), underscoring the need for rigorous on-target validation of dual-axis small molecules.

In summary, small molecules directly address delivery constraints intrinsic to antibody modalities while introducing tunable pharmacology and oral feasibility; success will depend on consolidating structural on-target validation, biomarker-driven pharmacodynamics and robust clinical efficacy that clearly differentiates these agents from established antibody checkpoints (134, 192). Near-term readouts from the programs listed in **Table 4** will determine whether oral PD-1/PD-L1 agents merit broader integration as complementary modalities.

**Table 4:** Clinical snapshot – small-molecule PD-1/PD-L1 inhibitors.

Program	Target/mechanism	Phase/status	Evidence summary	Identifier(s)
INCB086550	PD−L1 small−molecule inhibitor; dimerization/internalization	Early clinical (FiH/phase I)	Target−engagement signatures and immune pharmacodynamics demonstrated in translational studies with early human read−outs	(209)
Evixapodlin (GS−4224)	Oral PD−L1 inhibitor	Phase I	First−in−human trial reported PD−L1 engagement and expected immune PD	(210); NCT04049617
MAX−10181	Oral PD−L1 inhibitor	Phase I	Ongoing early−phase evaluation; limited public detail	(211); NCT04122339
CA−170	Oral PD−L1/VISTA modulator (putative)	Phase I/II (historical)	Early studies focused on safety; independent assays questioned direct PD−(L)1 inhibition	(212, 213); NCT02812875

No approved small−molecule PD-1/PD-L1 inhibitors as of 2025; programs emphasize on−target structural validation and biomarker−led pharmacodynamics.

## 7 Peptides and macrocycles targeting PD−1/PD−L1: compact ligands for difficult PPIs

### 7.1 Mechanistic rationale and emerging peptide/macrocycle formats

**Figure 9:**
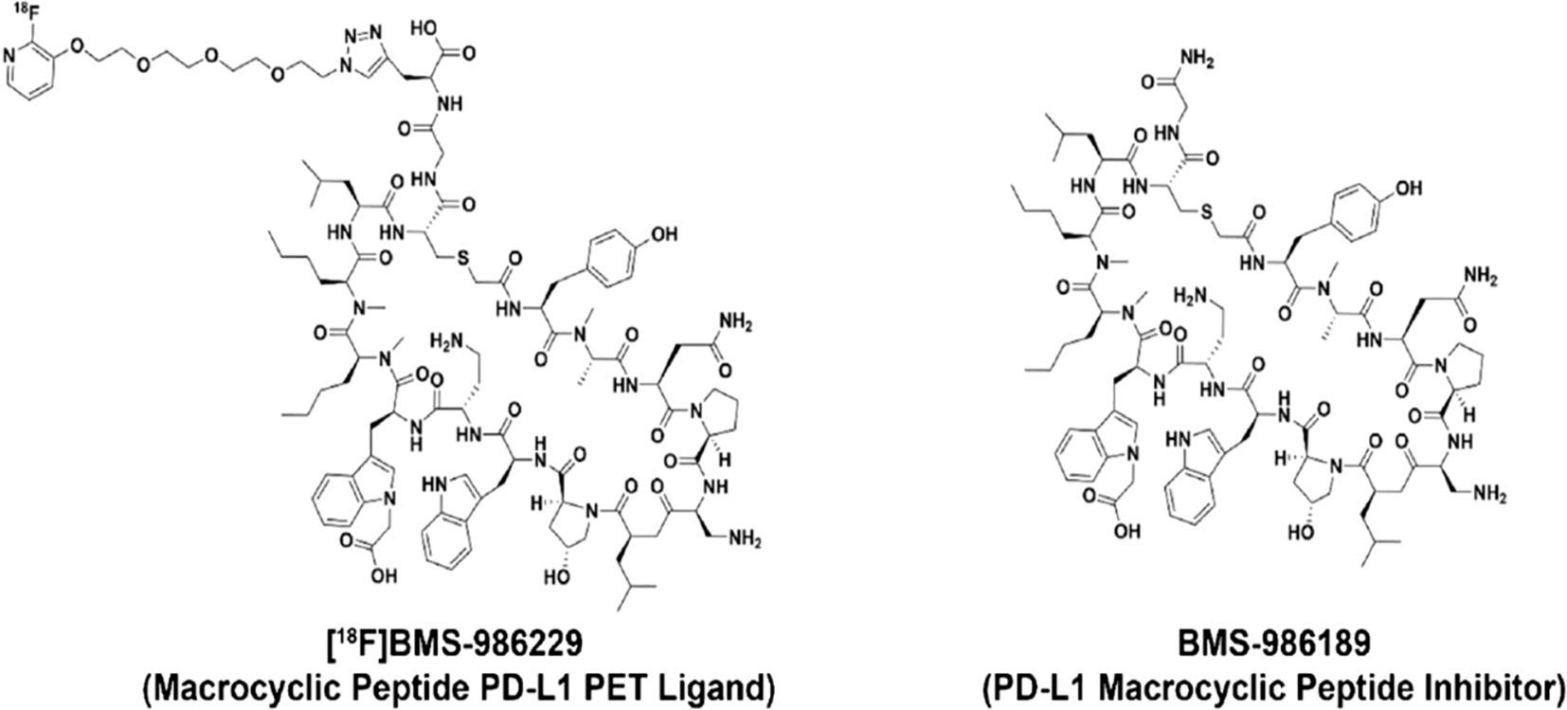
Structures of the macrocyclic PD-L1 inhibitor BMS-986189 and the PET radioligand [^18F]BMS-986229. Side-by-side macrocyclic peptide structures showing the shared scaffold for PD-L1 binding and the fluorine-18–labeled side chain on [^18F]BMS-986229 used for PET imaging.

**Figure 10:**
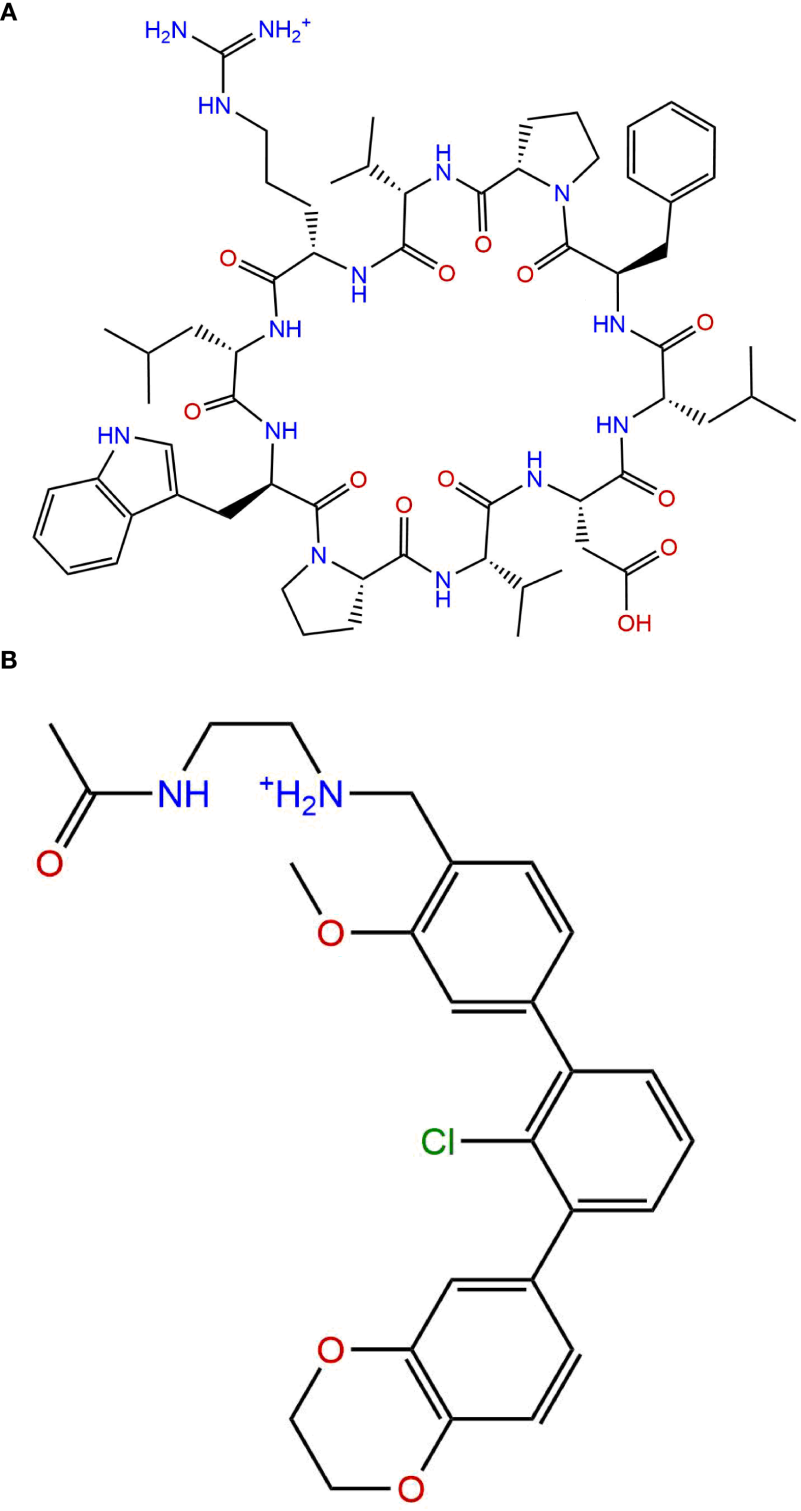
Compared structures of macrocycle and small-molecule ligands. **(A)** Structure of a macrocyclic peptide PD-L1 inhibitor reported by Sun et al. (IC_50_ = 7420 nM); **(B)** Structure of a rigid terphenyl-based small-molecule PD-L1 inhibitor reported by Muszak et al. (IC_50_ = 0.82 nM), references in text. IC_50_, half-maximal inhibitory concentration; lower IC_50_ values indicate higher potency. In this example, the small-molecule inhibitor in **(B)** is approximately 9,000-fold more potent than the macrocyclic peptide in **(A)**.

Peptides, and especially macrocyclic peptides (see **Figure 9**), offer geometrically adaptable contact surfaces capable of engaging the relatively flat PD-1/PD-L1 interface, where classical small-molecule drugs often struggle to achieve sufficient affinity and selectivity (214). Their relatively large, conformational pre-organized binding surfaces can mimic protein epitopes and more fully cover the shallow, hydrophobic PD-L1 patch than typical small molecules, which facilitates high-affinity engagement of this difficult protein–protein interface (215–217). Macrocyclization increases conformational pre-organization, proteolytic stability and cellular activity versus linear congeners, as shown for cyclic PD-L1 inhibitors that deliver multi-fold gains in PD-1/PD-L1 blockade and *in vivo* antitumor efficacy. Mechanistically, most macrocyclic peptides bind PD-L1 within hydrophobic pockets, occluding the PD-1-binding surface and preventing complex formation (218). Certain molecules can simultaneously disrupt PD-L1 interactions with PD-1 and with CD80 in cis, which may amplify immune activation (219). Alongside direct blockers, “degrading” strategies have emerged: stapled peptide PROTAC constructs drive PD-L1 depletion and reduce PD-L1 levels more effectively than the reference small-molecule inhibitor BMS-8 in cell systems (220). Consistent data show that lowering membrane PD-L1 is accompanied by enhanced T-cell activity, aligning with the pharmacodynamics rationale for targeted down-regulation (221). A parallel route employs PD-1-directed peptide ligands, rationally derived from PD-L1 contact regions and validated biophysically (SPR/ELISA) and in functional cellular assays, demonstrating targeted interception of the interface from the opposite side (214). Representative peptide and macrocycle programs are summarized in **Table 5**, and a structural comparison with a rigid small-molecule PD-L1 inhibitor is shown in **Figure 10**.

**Table 5:** Peptides & macrocycles – PD-1/PD-L1 inhibitors.

Program/tool	Type	Phase/status	Evidence summary	Identifier(s)
[18F]BMS−986229	Macrocyclic PD−L1 PET radioligand	Clinical PET initiated (imaging)	Competitive target−engagement PET read−outs and dosimetry shown in non−human primates; clinical imaging studies initiated	(223, 224)
BMS−986189	Macrocyclic PD−L1 inhibitor	Manufacturing readiness (process)	Scalable near−GMP process development (hundreds of grams API), stereocontrol and purity established	(222)
pAC65	Potent macrocyclic PD−L1 inhibitor (preclinical)	Preclinical	Dual interference with PD−L1:PD−1 and PD−L1:CD80 (cis) demonstrated; species−specificity limits translation	(219)

### 7.2 Developability, comparison with small molecules, and translational outlook

From a developability standpoint, PD-L1-focused macrocycles have progressed from concept to manufacturing-scale synthesis: process development has been reported for the macrocyclic inhibitor BMS-986189 at the hundreds-of-grams active-pharmaceutical-ingredient scale with solutions for stereocontrol and purity (222). The translational trajectory is further supported by macrocyclic PD-L1 radioligand tracers such as [18F]BMS-986229 (**Figure 9**): competitive target-engagement readouts and whole-body dosimetry have been demonstrated in non-human primates, and clinical positron emission tomography (PET) studies with this ligand have been initiated (223–225). Clinically, the field remains early: no peptide PD-1/PD-L1 inhibitors are approved, yet readiness for clinical formats is evidenced by PD-L1 radioligand imaging and the ability to scale macrocycles to pharmaceutical quality, whereas potent exemplars (for example, pAC65) still face preclinical species-specificity barriers (219, 222, 223).

In contrast to peptide macrocycles, small-molecule PD-L1 inhibitors are typically more rigid: widely used biphenyl and terphenyl series possess a core with restricted degrees of freedom, and ortho-substitution on one rings lock its orientation, favors ligand-induced PD-L1 dimerization snd enables very high affinity binding (204). Across published PD-L1 ligands, macrocyclic peptides usually span micromolar to high-nanomolar half-maximal inhibitory concentrations, whereas optimized small molecules can reach low-nanomolar or even picomolar potency; for example, macrocycles reported by Sun and colleagues are approximately 10^3^ to 10^4^ times less potent than terphenyl scaffolds described by Muszak and colleagues (204, 226). A representative structural comparison of a macrocyclic peptide PD-L1 inhibitor and a rigid terphenyl-based small-molecule PD-L1 inhibitor is shown in **Figure 10**.

Against this backdrop, peptide and macrocyclic ligands offer several physicochemical and pharmacologic advantages relative to small molecules. They are well suited to large and relatively flat protein-protein interfaces where key interaction hotspots are spatially dispersed, they can be engineered for modular conjugation or local delivery formats, and they often follow more predictable metabolic routes than classical xenobiotic small molecules (227). Macrocyclization further improves membrane permeability and resistance to proteolysis compared with linear peptides, which supports continued interest in macrocycles as an intermediate between large biologics and conventional small molecules (228). However, these advantages come at the cost of generally poor oral bioavailability, limited metabolic stability and lower intrinsic potency, so that chemical measures such as cyclization or stapling and parenteral administration are usually required (218, 229, 230).

Highly potent small molecules, in turn, maximize binding affinity but are more prone to off-target interactions with other proteins and to the formation of potentially toxic metabolites; very low, subnanomolar to picomolar inhibitory concentrations can narrow the therapeutic window, because toxic doses may be reached at relatively low systemic concentrations even though short half-lives partly mitigate overdose risk (231). From a chemistry standpoint, complex multistep synthetic routes can also render some small-molecule series more expensive to develop at scale (231).

With respect to immunogenicity, pharmacovigilance analyses suggest that peptides and macrocycles may be somewhat more predisposed to eliciting allergic reactions than small molecules, although patient-specific factors remain dominant determinants (232). These complementary profiles have motivated interest in combination strategies in which monoclonal antibodies are paired with peptide or small-molecule PD-1/PD-L1 modulators, for example by using peptides or macrocycles for imaging or local modulation while antibodies provide sustained systemic blockade (233).

No approved peptide/macrocyclic PD-1/PD-L1 therapeutics as of 2025; clinical activity to date centers on imaging tracers and manufacturing readiness rather than therapeutic efficacy endpoints. Looking ahead, three avenues are prominent: (i) further optimization of macrocycles guided by PD-L1 structural data and interaction maps to lower polarity while maintaining affinity (218); (ii) development of degrading and multimodal peptide constructs (PROTAC or conjugates) to achieve sustained PD-L1 suppression (220); and (iii) integration with local delivery and PET tracers to personalize dosing and patient selection (224, 225).

## 8 Decoy receptors and aptamers for PD−1/PD−L1: exotic avenues, constrained progress

### 8.1 PD−1 decoys (“traps”)

Over the past decade, a set of less established approaches has emerged alongside reduced-size antibody scaffolds and small molecules, most notably decoy receptors built from soluble PD-1 ectodomains and nucleic acid aptamers against PD-L1 (**Table 6**). These modalities were conceived to bypass limitations of IgG formats and classical medicinal chemistry by offering alternative geometries and manufacturability routes (234). Development has been steady but limited relative to antibodies, with few human efficacy signals to date (235). Conceptually, PD-1 decoy receptors and PD-L1 aptamers are positioned as non-antibody PD-1/PD-L1 blockers that can support alternative manufacturing, imaging and local-delivery solutions in settings where full-length monoclonal antibodies or small molecules are constrained by size, tissue penetration or immunogenicity (16, 234, 236).

**Table 6:** Representative PD-1/PD-L1 decoy receptors and aptamers (Preclinical stage).

Modality	Construct/target	Main application	DOI
Decoy receptor	High-affinity PD-1 ectodomain mutant “463”	Soluble PD-1/PD-L1 ligand trapping	(15)
Decoy receptor	Soluble PD-1 fusion with enhanced PD-L1/PD-L2 binding	Systemic decoy to overcome PD-1 resistance	(244)
Aptamer	Anti–PD-1 PEGylated DNA aptamer	Systemic PD-1 checkpoint inhibition	(245)
Aptamer	Antagonistic PD-L1 DNA aptamer (aptPD-L1)	Systemic PD-L1 checkpoint inhibition	(246)
Aptamer–NP	PD-L1 aptamer–hafnium oxide NP (Hf@ICG-Apt)	PD-L1-targeted NIR imaging and radiosensitization	(247)

Soluble PD-1 decoys can sequester PD-L1/PD-L2 and thereby interrupt PD-1 signaling without using an antibody paratope (**Table 6**). Proof-of-concept includes a plant-produced PD-1-Fc that blocks PD-1/PD-L1 *in vitro*, underscoring manufacturability in non-mammalian systems and the feasibility of the decoy geometry (237). Mechanistic precedent also comes from high-affinity PD-1 variants that exhibited greater antitumor activity than anti-PD-L1 antibodies in specific murine models and dosing regimens, and that served as immuno-PET probes, illustrating high-avidity trapping of PD-L1 *in vivo* (234). By contrast, a clinical pilot of the PD-1-engaging ligand-based decoy AMP-224 (PD-L2-Fc), in refractory metastatic colorectal cancer was tolerable but produced no objective responses, highlighting the translational gap (235).

### 8.2 PD−L1 aptamers

Aptamers are short single-stranded DNA or RNA ligands generated by systematic evolution of ligands by exponential enrichment (SELEX) and can be chemically modified and conjugated with ease, providing high affinity with low intrinsic immunogenicity (236). Recent selections yielded new DNA aptamers to PD-L1 with nanomolar affinity and functional blockade in cell systems, confirming tractable discovery and on-target activity (238). Pharmacokinetic limitations, notably rapid renal clearance, can be mitigated by increasing hydrodynamic size, for example via albumin conjugation, which improved *in vivo* antitumor efficacy of a PD-L1 aptamer (239). Aptamer-guided nanocarriers have also been engineered to respond to radiotherapy, enhancing reactive oxygen species production and macrophage reprogramming in tumor models (240). Beyond discovery tools, antagonistic aptamers such as PL1 restored T-cell function and inhibited growth of PD-L1-positive tumors in mice, reinforcing biological validity (241).

#### 8.2.1 Why progress has remained limited after approximately ten years

(i) Unlike antibodies, decoy receptors and aptamers have not produced positive late-phase oncology trials; early decoy formats such as AMP-224 yielded no objective responses in refractory metastatic colorectal cancer (235). (ii) Nucleic acid aptamers suffer rapid renal clearance and nuclease degradation, necessitating chemical protection and size augmentation, for example via albumin conjugation, while manufacturing and translational pathways, including plant-produced Fc fusions, are less mature than IgG platforms (237, 239). (iii) Target-network complexity: PD-L1 participates in cis interactions, for example PD-L1:CD80, so format-specific perturbations risk unintended network effects without careful engineering (219). Thus, decoy receptors and aptamers expand the conceptual toolkit for PD-1/PD-L1 by offering non-antibody routes to blockade or delivery, yet, as of 2025, they remain minor modalities pending convincing clinical efficacy that surpasses, or at least complements, established antibody standards (235). For the reader’s convenience, all classes of promising therapeutic molecules described in the text are summarized in the **Supplementary Table S7** and compared with mAbs. Examples of the most promising alternatives as of 2025, based on combined mechanistic rationale, maturity and translational trajectory, are summarized in **Supplementary Table S8**.

From a translational standpoint, decoy receptors and aptamers are also candidates for localized delivery and diagnostic imaging rather than systemic antagonism alone (16). High affinity soluble PD-1 ectodomains and related decoy constructs are conceptually well suited for vectorized or matrix-anchored formats that concentrate checkpoint blockade within the tumor microenvironment, although this remains largely at the preclinical or theoretical stage for PD-1/PD-L1 (16). By contrast, PD-L1-targeted aptamers have already been used to generate PET probes that noninvasively report PD-L1 expression dynamics *in vivo* (242) and dual PET/near-infrared fluorescence probes based on “chemical antibody-aptamer” designs (243). These and related constructs can be coupled to nanoparticle or radionuclide carriers, highlighting a potential niche for aptamer-based imaging and locally enriched delivery rather than standalone systemic therapy (16).

## 9 Conclusion

Antibodies targeting PD-1/PD-L1 remain the mainstay of immunotherapy, setting the reference for survival and durability of response across multiple tumor types. Yet mAbs have inherent constraints, including incomplete intratumoral penetration, long half-life with limited real-time toxicity control, immune-related adverse events and resistance within an immunosuppressive microenvironment. These limitations motivate a diversified pipeline of alternative PD-1/PD-L1-targeting formats rather than diminish the value of antibodies.

Within antibody-based strategies, Fc-modified antibodies, bispecifics, reduced-size scaffolds and locally delivered or gene-encoded fragments follow distinct trajectories. Bispecific antibodies integrate PD-1/PD-L1 blockade with co-inhibition or context-dependent co-stimulation, increasing functional avidity where co-expression is present. Reduced-size antibody scaffolds improve diffusion and formatting flexibility, with Fc or albumin fusion controlling pharmacokinetics and routes of administration. Locally delivered or gene-encoded fragments can create high local ligand gradients. Late-phase validation is still maturing but delineates areas of clinical promise.

Compared with antibodies, small molecules that induce PD-L1 dimerization and shield the PD-1 interface offer oral dosing, penetration of fibrotic stroma and poorly perfused regions, and a short, controllable half-life. Their success will hinge on robust structural on-target validation, biomarker-guided pharmacodynamics and convincing clinical efficacy comparable to accepted antibody standards. Additional approaches, including decoy receptors and aptamers, broaden the toolkit for blockade and delivery, although they remain minor owing to pharmacokinetic and manufacturing constraints and limited clinical signals. Taken together, these observations support selective adoption of complementary formats in defined niches; candidates with the most favorable 2025 profile are summarized in **Supplementary Table S8**.

Effective PD-1/PD-L1 therapy is increasingly about fit for context. Patient stratification should integrate spatial co-expression, antigen presentation, microenvironmental composition and tissue accessibility. Trial design should incorporate measurable proxies of exposure and action, including PD-1/PD-L1-oriented pharmacodynamic markers and spatial distribution metrics. Combinations should be assembled so that valence, size and site of action complement one another. In sum, antibodies retain a foundational role, while the diversity of alternative scaffolds and small molecules offers routes to overcome built-in physical and biological limits. As late-phase confirmation accrues, a modular and personalized PD-1/PD-L1 landscape is likely to emerge.
