# Supplementary material for: Beyond monoclonal antibodies: constraints and the case for alternative PD-1/PD-L1-targeting formats
Source: Front Immunol. 2025 Dec 17;16:1729468. doi: 10.3389/fimmu.2025.1729468 (PMC12753384; doi:10.3389/fimmu.2025.1729468)
Supplement: Supplementary file 1 [file Table1.docx]

**Supplementary Table S1.** Glossary of abbreviations used in the article

| Abbreviation | Definition |
| --- | --- |
| AAV8 | Adeno-associated virus serotype 8 |
| APC | Antigen-presenting cell |
| CC′ loop | Loop between C and C′ β-strands of the IgV fold |
| DART | Dual-Affinity Re-Targeting (bispecific antibody format) |
| dMMR | Deficient mismatch repair |
| Fab | Antigen-binding fragment |
| GFCC′ sheet | β-sheet comprising G, F, C and C′ strands (IgV fold) |
| GM-CSF | Granulocyte-macrophage colony-stimulating factor |
| HNSCC | Head and neck squamous cell carcinoma |
| HSV-1 | Herpes simplex virus type 1 |
| hNIS | Human sodium/iodide symporter |
| ICI | Immune checkpoint inhibitor(s) |
| IgV | Immunoglobulin variable-like domain (Ig-like V-type) |
| IL-12 | Interleukin-12 |
| ITIM | Immunoreceptor tyrosine-based inhibitory motif |
| ITSM | Immunoreceptor tyrosine-based switch motif |
| LPS | Lipopolysaccharide |
| mCRC | Metastatic colorectal cancer |
| MC38 | Murine colon adenocarcinoma cell line MC38 |
| MSI-H | Microsatellite instability-high |
| MST | Microscale thermophoresis |
| NSCLC | Non-small-cell lung cancer |
| PPI | Protein–protein interaction |
| QW / Q2W | Once weekly / once every two weeks |
| RCT | Randomized controlled trial |
| RM-NPC | Recurrent/metastatic nasopharyngeal carcinoma |
| ROS | Reactive oxygen species |
| scFv | Single-chain variable fragment |
| sdAb | Single-domain antibody |
| SELEX | Systematic evolution of ligands by exponential enrichment |
| SH2 | Src homology 2 (domain) |
| SHP-1 / SHP-2 | Src homology region-2-containing phosphatase-1 / -2 |
| TCR | T-cell receptor |
| Tex | Exhausted T cell |
| Th1 | T helper 1 cell |
| VH | Variable domain of the heavy chain |
| VHH | Variable domain of heavy-chain-only antibody (‘nanobody’) |
