# Supplementary material for: Beyond monoclonal antibodies: constraints and the case for alternative PD-1/PD-L1-targeting formats
Source: Front Immunol. 2025 Dec 17;16:1729468. doi: 10.3389/fimmu.2025.1729468 (PMC12753384; doi:10.3389/fimmu.2025.1729468)
Supplement: Supplementary file 2 [file Table2.docx]

**Supplementary Table S2.** Induction and cellular sources of PD-L1/PD-L2 and clinical correlates of sPD-L1

| Topic | Summary | Representative sources |
| --- | --- | --- |
| PD-L1 on APCs | Induced on dendritic cells and macrophages by LPS and GM-CSF | (1-4) |
| PD-L1 on T/B cells | Upregulated on T and B lymphocytes following TCR activation | (5-8) |
| PD-L1 on tumor cells | Upregulated by interferon-γ and inflammatory cues from naive/Th1 CD4⁺, memory T cells, CD8⁺, NK, dendritic cells and B cells | (9, 10) |
| PD-L2 expression | Detected on dendritic-cell subsets and specific tumor lines | (11-14) |
| sPD-L1 | Higher serum levels correlate with poorer response to PD-(L)1 blockade; biology remains incompletely defined | (15-18) |

**References:**

1. Peng Q, Qiu X, Zhang Z, Zhang S, Zhang Y, Liang Y, et al. Pd-L1 on Dendritic Cells Attenuates T Cell Activation and Regulates Response to Immune Checkpoint Blockade. *Nat Commun* (2020) 11(1):4835. Epub 2020/09/26. doi: 10.1038/s41467-020-18570-x.

2. Rong QX, Wang F, Guo ZX, Hu Y, An SN, Luo M, et al. Gm-Csf Mediates Immune Evasion Via Upregulation of Pd-L1 Expression in Extranodal Natural Killer/T Cell Lymphoma. *Mol Cancer* (2021) 20(1):80. Epub 2021/05/31. doi: 10.1186/s12943-021-01374-y.

3. Lellahi SM, Azeem W, Hua Y, Gabriel B, Paulsen Rye K, Reikvam H, et al. Gm-Csf, Flt3-L and Il-4 Affect Viability and Function of Conventional Dendritic Cell Types 1 and 2. *Front Immunol* (2022) 13:1058963. Epub 2023/01/31. doi: 10.3389/fimmu.2022.1058963.

4. Yonemitsu K, Pan C, Fujiwara Y, Miyasato Y, Shiota T, Yano H, et al. Gm-Csf Derived from the Inflammatory Microenvironment Potentially Enhanced Pd-L1 Expression on Tumor-Associated Macrophages in Human Breast Cancer. *Sci Rep* (2022) 12(1):12007. Epub 2022/07/15. doi: 10.1038/s41598-022-16080-y.

5. Pulko V, Harris KJ, Liu X, Gibbons RM, Harrington SM, Krco CJ, et al. B7-H1 Expressed by Activated Cd8 T Cells Is Essential for Their Survival. *J Immunol* (2011) 187(11):5606-14. Epub 2011/10/26. doi: 10.4049/jimmunol.1003976.

6. Karwacz K, Bricogne C, MacDonald D, Arce F, Bennett CL, Collins M, et al. Pd-L1 Co-Stimulation Contributes to Ligand-Induced T Cell Receptor Down-Modulation on Cd8+ T Cells. *EMBO Mol Med* (2011) 3(10):581-92. Epub 2011/07/09. doi: 10.1002/emmm.201100165.

7. Latchman YE, Liang SC, Wu Y, Chernova T, Sobel RA, Klemm M, et al. Pd-L1-Deficient Mice Show That Pd-L1 on T Cells, Antigen-Presenting Cells, and Host Tissues Negatively Regulates T Cells. *Proc Natl Acad Sci U S A* (2004) 101(29):10691-6. Epub 2004/07/14. doi: 10.1073/pnas.0307252101.

8. Liu R, Li HF, Li S. Pd-1-Mediated Inhibition of T Cell Activation: Mechanisms and Strategies for Cancer Combination Immunotherapy. *Cell Insight* (2024) 3(2):100146. Epub 2024/03/01. doi: 10.1016/j.cellin.2024.100146.

9. Qian J, Wang C, Wang B, Yang J, Wang Y, Luo F, et al. The Ifn-Gamma/Pd-L1 Axis between T Cells and Tumor Microenvironment: Hints for Glioma Anti-Pd-1/Pd-L1 Therapy. *J Neuroinflammation* (2018) 15(1):290. Epub 2018/10/20. doi: 10.1186/s12974-018-1330-2.

10. Imai Y, Chiba T, Kondo T, Kanzaki H, Kanayama K, Ao J, et al. Interferon-Gamma Induced Pd-L1 Expression and Soluble Pd-L1 Production in Gastric Cancer. *Oncol Lett* (2020) 20(3):2161-8. Epub 2020/08/13. doi: 10.3892/ol.2020.11757.

11. Loke P, Allison JP. Pd-L1 and Pd-L2 Are Differentially Regulated by Th1 and Th2 Cells. *Proc Natl Acad Sci U S A* (2003) 100(9):5336-41. Epub 2003/04/17. doi: 10.1073/pnas.0931259100.

12. Yearley JH, Gibson C, Yu N, Moon C, Murphy E, Juco J, et al. Pd-L2 Expression in Human Tumors: Relevance to Anti-Pd-1 Therapy in Cancer. *Clin Cancer Res* (2017) 23(12):3158-67. Epub 2017/06/18. doi: 10.1158/1078-0432.CCR-16-1761.

13. Wang Y, Du J, Gao Z, Sun H, Mei M, Wang Y, et al. Evolving Landscape of Pd-L2: Bring New Light to Checkpoint Immunotherapy. *Br J Cancer* (2023) 128(7):1196-207. Epub 2022/12/16. doi: 10.1038/s41416-022-02084-y.

14. Sun Y, Yang J, Chen Y, Guo Y, Xiong J, Guo X, et al. Pd-L2 Expression in Breast Cancer Promotes Tumor Development and Progression. *J Immunol Res* (2024) 2024:3145695. Epub 2024/07/10. doi: 10.1155/2024/3145695.

15. Oh SY, Kim S, Keam B, Kim TM, Kim DW, Heo DS. Soluble Pd-L1 Is a Predictive and Prognostic Biomarker in Advanced Cancer Patients Who Receive Immune Checkpoint Blockade Treatment. *Sci Rep* (2021) 11(1):19712. Epub 2021/10/07. doi: 10.1038/s41598-021-99311-y.

16. Perez-Picazo SE, Martinez-Morales P, Conde-Rodriguez I, Reyes-Leyva J, Vallejo-Ruiz V. High Serum Levels of Soluble Pd‑1 and Pd‑L1 Are Associated with Advanced Clinical Stages in Patients with Cervical Cancer. *Biomed Rep* (2025) 22(4):70. Epub 2025/02/28. doi: 10.3892/br.2025.1948.

17. Kushlinskii NE, Kovaleva OV, Gratchev AN, Alferov AA, Kuzmin YB, Sokolov NY, et al. Assessing the Clinical Relevance of Soluble Pd-1 and Pd-L1: A Multi-Cohort Study across Diverse Tumor Types and Prognostic Implications. *Biomedicines* (2025) 13(2). Epub 2025/02/26. doi: 10.3390/biomedicines13020500.

18. Al-Khazaleh AK, Alsherbiny MA, Chang D, Munch G, Bhuyan DJ. Investigating the Cellular Responses to Combined Nisin and Urolithin B Treatment (7:3) in Hkb-11 Lymphoma Cells. *Int J Mol Sci* (2025) 26(15). Epub 2025/08/14. doi: 10.3390/ijms26157369.
