# Supplementary material for: Beyond monoclonal antibodies: constraints and the case for alternative PD-1/PD-L1-targeting formats
Source: Front Immunol. 2025 Dec 17;16:1729468. doi: 10.3389/fimmu.2025.1729468 (PMC12753384; doi:10.3389/fimmu.2025.1729468)
Supplement: Supplementary file 3 [file Table3.docx]

**Supplementary Table S3.** Residue-level and conformational notes for PD-1 upon ligand binding

| Region/feature | Perturbed residues (PD-1) | Note and sources |
| --- | --- | --- |
| CC′ loop and GFCC′ sheet | Phe63, Asn66, Tyr68, Arg69, Ser73, Thr76, Ala80, Asp85, Gln88, Arg112, Thr120, Leu122, Cys123, Gly124, Ala125, Ala132, Gln133, Ser137, Thr59, Ser60, Glu61, Leu128, Lys131; centered on Gln75 | CC′ loop remodeling and interface formation drive complex stabilization; PD-1:PD-L1 interaction is relatively entropy-driven, PD-1:PD-L2 shows a larger enthalpic component (1, 2);  Human PD-1:PD-L1 (X-ray, complex) - (3);  Murine PD-1:human PD-L1(1); Murine PD-1:murine PD-L2 (4);  PD-1:PD-L1 vs PD-1:PD-L2 (2,5,6);  PDB 3RRQ (human PD-1 ectodomain) (7) |

**References:**

1. Lin DY, Tanaka Y, Iwasaki M, Gittis AG, Su HP, Mikami B, et al. The Pd-1/Pd-L1 Complex Resembles the Antigen-Binding Fv Domains of Antibodies and T Cell Receptors. *Proc Natl Acad Sci U S A* (2008) 105(8):3011-6. Epub 2008/02/22. doi: 10.1073/pnas.0712278105.

2. Cheng X, Veverka V, Radhakrishnan A, Waters LC, Muskett FW, Morgan SH, et al. Structure and Interactions of the Human Programmed Cell Death 1 Receptor. *J Biol Chem* (2013) 288(17):11771-85. Epub 2013/02/19. doi: 10.1074/jbc.M112.448126.

3. Zak KM, Kitel R, Przetocka S, Golik P, Guzik K, Musielak B, et al. Structure of the Complex of Human Programmed Death 1, Pd-1, and Its Ligand Pd-L1. *Structure* (2015) 23(12):2341-8. Epub 2015/11/26. doi: 10.1016/j.str.2015.09.010.

4. Lazar-Molnar E, Yan Q, Cao E, Ramagopal U, Nathenson SG, Almo SC. Crystal Structure of the Complex between Programmed Death-1 (Pd-1) and Its Ligand Pd-L2. *Proc Natl Acad Sci U S A* (2008) 105(30):10483-8. Epub 2008/07/22. doi: 10.1073/pnas.0804453105.

5. Hong Y, Feng Y, Sun H, Zhang B, Wu H, Zhu Q, et al. Tislelizumab Uniquely Binds to the Cc' Loop of Pd-1 with Slow-Dissociated Rate and Complete Pd-L1 Blockage. *FEBS Open Bio* (2021) 11(3):782-92. Epub 2021/02/03. doi: 10.1002/2211-5463.13102.

6. Horita S, Nomura Y, Sato Y, Shimamura T, Iwata S, Nomura N. High-Resolution Crystal Structure of the Therapeutic Antibody Pembrolizumab Bound to the Human Pd-1. *Sci Rep* (2016) 6:35297. Epub 2016/10/14. doi: 10.1038/srep35297.

7. 3rrq: Human Pd-1 Ectodomain (Apo): RCSB Protein Data Bank. Protein Data Bank:[Available from: https://doi.org/10.2210/pdb3RRQ/pdb.
