# Supplementary material for: Beyond monoclonal antibodies: constraints and the case for alternative PD-1/PD-L1-targeting formats
Source: Front Immunol. 2025 Dec 17;16:1729468. doi: 10.3389/fimmu.2025.1729468 (PMC12753384; doi:10.3389/fimmu.2025.1729468)
Supplement: Supplementary file 4 [file Table4.docx]

**Supplementary Table S4.** Structural resources for PD-1 and its ligand complexes

| PDB ID | Complex | Note | References |
| --- | --- | --- | --- |
| 3RRQ | PD-1 ectodomain (apo) | Representative apo structure of PD-1 | (1) |
| 2M2D | PD-1 ectodomain (apo) | Additional apo entry | (2) |
| 6UMT | PD-1:PD-L1 complex | Complex structure of PD-1 with PD-L1 | (3-5) |
| 4ZQK | PD-1:PD-L2 complex | Complex structure of PD-1 with PD-L2 | (3, 4) |
| 5JXE | PD-1 : pembrolizumab Fab | Therapeutic antibody complex | (6, 7) |
| 5WT9 | PD-1 : nivolumab Fab | Therapeutic antibody complex | (8, 9) |
| 3BIK | PD-1:PD-L1 complex | Murine PD-1 with human PD-L1 (seminal architecture) | (10, 11) |
| - | UniProt Q15116 | Additional entries via UniProt structure portal (URL per journal policy) | - |

**References:**

1. 3rrq: Human Pd-1 Ectodomain (Apo): RCSB Protein Data Bank. Protein Data Bank:[Available from: <https://doi.org/10.2210/pdb3RRQ/pdb>.

2. Cheng X, Veverka V, Radhakrishnan A, Waters LC, Muskett FW, Morgan SH, et al. Structure and Interactions of the Human Programmed Cell Death 1 Receptor. *J Biol Chem* (2013) 288(17):11771-85. Epub 2013/02/19. doi: 10.1074/jbc.M112.448126.

3. Zak KM, Kitel R, Przetocka S, Golik P, Guzik K, Musielak B, et al. Structure of the Complex of Human Programmed Death 1, Pd-1, and Its Ligand Pd-L1. *Structure* (2015) 23(12):2341-8. Epub 2015/11/26. doi: 10.1016/j.str.2015.09.010.

4. Tang S, Kim PS. A High-Affinity Human Pd-1/Pd-L2 Complex Informs Avenues for Small-Molecule Immune Checkpoint Drug Discovery. *Proc Natl Acad Sci U S A* (2019) 116(49):24500-6. Epub 2019/11/16. doi: 10.1073/pnas.1916916116.

5. 6umt: Pd-1 Bound to Pd-L2: RCSB Protein Data Bank. Protein Data Bank:[Available from: <https://doi.org/10.2210/pdb6umt/pdb>.

6. Na Z, Yeo SP, Bharath SR, Bowler MW, Balikci E, Wang CI, et al. Structural Basis for Blocking Pd-1-Mediated Immune Suppression by Therapeutic Antibody Pembrolizumab. *Cell Res* (2017) 27(1):147-50. Epub 2016/06/22. doi: 10.1038/cr.2016.77.

7. 5jxe: Pd-1 Bound to Pembrolizumab Fab: RCSB Protein Data Bank. Protein Data Bank:[Available from: <https://doi.org/10.2210/pdb5jxe/pdb>.

8. Tan S, Zhang H, Chai Y, Song H, Tong Z, Wang Q, et al. An Unexpected N-Terminal Loop in Pd-1 Dominates Binding by Nivolumab. *Nat Commun* (2017) 8:14369. Epub 2017/02/07. doi: 10.1038/ncomms14369.

9. 5wt9: Pd-1 Bound to Nivolumab Fab: RCSB Protein Data Bank. Protein Data Bank:[Available from: <https://doi.org/10.2210/pdb5wt9/pdb>.

10. Lin DY, Tanaka Y, Iwasaki M, Gittis AG, Su HP, Mikami B, et al. The Pd-1/Pd-L1 Complex Resembles the Antigen-Binding Fv Domains of Antibodies and T Cell Receptors. *Proc Natl Acad Sci U S A* (2008) 105(8):3011-6. Epub 2008/02/22. doi: 10.1073/pnas.0712278105.

11. 3bik: Pd-1 Bound to Pd-L1 (Murine Pd-1, Human Pd-L1): RCSB Protein Data Bank. Protein Data Bank:[Available from: <https://doi.org/10.2210/pdb3bik/pdb>.
