# Supplementary material for: Beyond monoclonal antibodies: constraints and the case for alternative PD-1/PD-L1-targeting formats
Source: Front Immunol. 2025 Dec 17;16:1729468. doi: 10.3389/fimmu.2025.1729468 (PMC12753384; doi:10.3389/fimmu.2025.1729468)
Supplement: Supplementary file 5 [file Table5.docx]

**Supplementary Table S5.** Antibody–Antibody Combinations (ICI + ICI or immune-modulating Abs)

| Combination / target(s) | Indication / population | Key metric (summary) | Net effect | References |
| --- | --- | --- | --- | --- |
| ^★^PD‑1 + CTLA‑4 (nivolumab + ipilimumab) | Unresectable/metastatic melanoma, 1st line | 10‑year OS benefit sustained^†^; earlier readouts showed PFS/ORR gains | Deepest and most durable effect vs PD‑1 alone with ↑irAEs^†^ | NIVO+IPI (melanoma, 1L): (1, 2) Mechanistic note^¶^: (3) |
| ^★^PD‑1 + LAG‑3 (nivolumab + relatlimab) | Melanoma, 1st line | Improved PFS/OS vs PD‑1; more favorable irAE profile^†^ than PD‑1+CTLA‑4; advantage maintained in updates | Sustained benefit in longer follow‑up^†^ | (4-6) |
| PD‑(L)1 + TIGIT (tiragolumab + atezolizumab, etc.) | NSCLC, PD‑L1‑high, 1st line | CITYSCAPE: ↑ORR and PFS trend; later phase results mixed^‡§^ / context‑dependent | Promising but biomarker selection is key ^‡^ | CITYSCAPE (phase 2) (7); SKYSCRAPER-01 (8); SKYSCRAPER-02 (SCLC) (9) |
| PD‑1 + TIM‑3 (e.g., INCAGN02390 ± PD‑1) | Early solid‑tumor cohorts | Phase I: acceptable safety; biologic rationale for PD‑1 combinations | Conceptual synergy supported by early data | (10, 11) |

**Abbreviations:** 1L, first-line therapy; ATEZO, atezolizumab; INCAGN02390, anti–TIM-3 monoclonal antibody (Incyte); irAE(s), immune-related adverse event(s); IPI, ipilimumab; LAG-3, lymphocyte-activation gene 3; NSCLC, non-small cell lung cancer; ORR, objective response rate; OS, overall survival; PFS, progression-free survival; RELAT, relatlimab; SCLC, small cell lung cancer; TIGIT, T-cell immunoreceptor with Ig and ITIM domains; TIM-3, T-cell immunoglobulin and mucin-domain containing-3; TPS, tumor proportion score; TIRA, tiragolumab; NIVO, nivolumab.

^★^late-phase evidence with OS benefit (see references)

^†^ irAE profile: For NIVO+IPI there is a sustained OS advantage at the cost of higher irAE rates; NIVO+RELAT shows a more favorable toxicity profile while maintaining efficacy gains.

^‡^ Biomarker definitions: In CITYSCAPE/NSCLC, “PD-L1-high” corresponded to TPS ≥ 50% (per trial assays/protocols); PD-L1 thresholding remains critical for TIGIT combinations.

^§^ Evidence heterogeneity: TIGIT programs yield divergent PFS/OS results across indications (NSCLC vs SCLC) and study designs; interpretation should account for population and biomarker context.

^¶^ Mechanistic footnote: The cited PNAS data describe cellular mechanisms of CTLA-4+PD-1 synergy and do not substitute for clinical outcomes.

**References:**

1. Wolchok JD, Chiarion-Sileni V, Rutkowski P, Cowey CL, Schadendorf D, Wagstaff J, et al. Final, 10-Year Outcomes with Nivolumab Plus Ipilimumab in Advanced Melanoma. *N Engl J Med* (2025) 392(1):11-22. Epub 2024/09/17. doi: 10.1056/NEJMoa2407417.

2. Wolchok JD, Chiarion-Sileni V, Gonzalez R, Grob JJ, Rutkowski P, Lao CD, et al. Long-Term Outcomes with Nivolumab Plus Ipilimumab or Nivolumab Alone Versus Ipilimumab in Patients with Advanced Melanoma. *J Clin Oncol* (2022) 40(2):127-37. Epub 2021/11/25. doi: 10.1200/JCO.21.02229.

3. Wei SC, Anang NAS, Sharma R, Andrews MC, Reuben A, Levine JH, et al. Combination Anti-Ctla-4 Plus Anti-Pd-1 Checkpoint Blockade Utilizes Cellular Mechanisms Partially Distinct from Monotherapies. *Proc Natl Acad Sci U S A* (2019) 116(45):22699-709. Epub 2019/10/23. doi: 10.1073/pnas.1821218116.

4. Tawbi HA, Hodi FS, Lipson EJ, Schadendorf D, Ascierto PA, Matamala L, et al. Three-Year Overall Survival with Nivolumab Plus Relatlimab in Advanced Melanoma from Relativity-047. *J Clin Oncol* (2025) 43(13):1546-52. Epub 2024/12/13 20:06. doi: 10.1200/JCO.24.01124.

5. Lipson EJ, Stephen Hodi F, Tawbi H, Schadendorf D, Ascierto PA, Matamala L, et al. Nivolumab Plus Relatlimab in Advanced Melanoma: Relativity-047 4-Year Update. *Eur J Cancer* (2025) 225:115547. Epub 2025/06/14. doi: 10.1016/j.ejca.2025.115547.

6. Tawbi HA, Schadendorf D, Lipson EJ, Ascierto PA, Matamala L, Castillo Gutierrez E, et al. Relatlimab and Nivolumab Versus Nivolumab in Untreated Advanced Melanoma. *N Engl J Med* (2022) 386(1):24-34. Epub 2022/01/06. doi: 10.1056/NEJMoa2109970.

7. Cho BC, Abreu DR, Hussein M, Cobo M, Patel AJ, Secen N, et al. Tiragolumab Plus Atezolizumab Versus Placebo Plus Atezolizumab as a First-Line Treatment for Pd-L1-Selected Non-Small-Cell Lung Cancer (Cityscape): Primary and Follow-up Analyses of a Randomised, Double-Blind, Phase 2 Study. *Lancet Oncol* (2022) 23(6):781-92. Epub 2022/05/17. doi: 10.1016/S1470-2045(22)00226-1.

8. Skyscraper-01 (Nsclc, Pd-L1-High): Phase 3 Topline Result — Os/Pfs Not Met: Program update (topline report) (2025).

9. Rudin CM, Liu SV, Soo RA, Lu S, Hong MH, Lee JS, et al. Skyscraper-02: Tiragolumab in Combination with Atezolizumab Plus Chemotherapy in Untreated Extensive-Stage Small-Cell Lung Cancer. *J Clin Oncol* (2024) 42(3):324-35. Epub 2023/11/17. doi: 10.1200/JCO.23.01363.

10. Gutierrez ME, Tang SC, Powderly JD, 2nd, Balmanoukian AS, Hoyle PE, Dong Z, et al. First-in-Human Phase I Open-Label Study of the Anti-Tim-3 Monoclonal Antibody Incagn02390 in Patients with Select Advanced or Metastatic Solid Tumors. *Oncologist* (2025) 30(7). Epub 2025/07/09. doi: 10.1093/oncolo/oyaf144.

11. Cai L, Li Y, Tan J, Xu L, Li Y. Targeting Lag-3, Tim-3, and Tigit for Cancer Immunotherapy. *J Hematol Oncol* (2023) 16(1):101. Epub 2023/09/06. doi: 10.1186/s13045-023-01499-1.
