# Supplementary material for: Beyond monoclonal antibodies: constraints and the case for alternative PD-1/PD-L1-targeting formats
Source: Front Immunol. 2025 Dec 17;16:1729468. doi: 10.3389/fimmu.2025.1729468 (PMC12753384; doi:10.3389/fimmu.2025.1729468)
Supplement: Supplementary file 6 [file Table6.docx]

**Supplementary Table S6**. Antibody + Non-Antibody Modalities (small molecules/Fc engineering/bispecifics)

| Combination / target(s) | Indication / population | Key metric (summary) | Net effect | References |
| --- | --- | --- | --- | --- |
| ^★^PD‑1×VEGF bispecific (ivonescimab) | NSCLC, PD‑L1‑positive, 1st line | HARMONi‑2: PFS superior vs pembrolizumab; first approval in China (2024/2025) per The Lancet report; global positioning pending | System‑level gain via vessel normalization + PD‑1 blockade | (1-3) |
| Nivolumab + ruxolitinib (JAK1/2) | R/R cHL post‑CPI | Early clinical activity, manageable safety; translational support for CPI resensitisation | Proof‑of‑concept for combining JAK inhibition with CPI | (4-6) |
| Ciforadenant + atezolizumab (A2AR + PD‑L1) | RCC / solid tumors, early‑phase | Activity signal in “adenosine‑high” subsets; manageable safety | Translationally selected benefit | (7) |
| Emactuzumab (anti‑CSF1R) + atezolizumab | Multiple solid tumors, early‑phase | Biologic and safety signals; ↑CD8⁺ TIL in subsets | Feasible; rationale for myeloid‑targeted combos | (8) |
| ARRY‑382 (CSF1R small molecule) + pembrolizumab | Advanced solid tumors | Pharmacodynamic CSF1R suppression; limited clinical activity | Early‑phase feasibility; marginal efficacy | (9) |
| Pexidartinib (PLX3397) + pembrolizumab | Solid tumors (selected), early‑phase | Dose‑finding; TAM‑directed rationale | Phase 1-2 without mature survival readouts | (10); NCT02452424 |
| Eganelisib (IPI‑549; PI3Kγ) + PD‑(L)1 | MARIO‑1 & others; early‑phase | Acceptable safety; preliminary activity incl. PD‑(L)1‑experienced pts | Limited clinical activity to date | (11) |
| Navarixin (MK‑7123; CXCR1/2) + pembrolizumab | NSCLC; phase II | No OS benefit; safety acceptable | Context‑dependent, biomarker‑guided use needed | (12) |
| SX‑682 (CXCR1/2) + pembrolizumab | Early solid‑tumor cohorts | Early‑phase; ongoing | Feasible; awaiting efficacy | NCT03161431 |
| BMS‑986253 (anti‑IL‑8) + nivolumab ± ipilimumab | Advanced cancers | Phase I: safety and biomarker modulation | Biologic rationale; early‑phase | NCT03400332 |
| Cabozantinib + atezolizumab | mCRPC, phase III (CONTACT‑02) | ↑PFS; co-primary OS not met; no filing announced at data cut | Mixed late‑phase efficacy; no filing | (13, 14) |
| Platelet-targeted ABT-737 nanoparticles (cRGD-NP@A) + anti-PD-1 | Preclinical metastasis models | Selective ablation of tumor-associated platelets and enhanced anti-PD-1 efficacy without added systemic toxicity | Microenvironment-aware delivery to intensify checkpoint blockade | (15) |

**Abbreviations:** A2AR, adenosine A2A receptor; cHL, classical Hodgkin lymphoma; CPI, checkpoint inhibitor; FiH, first-in-human; mCRPC, metastatic castration-resistant prostate cancer; NSCLC, non-small-cell lung cancer; ORR, objective response rate; OS, overall survival; PFS, progression-free survival; RCC, renal cell carcinoma; TAM, tumour-associated macrophage; TIL, tumour-infiltrating lymphocyte.

^★^Eentries with late-phase evidence and/or regulatory action in 2024–2025.

**References:**

1. Xiong A, Wang L, Chen J, Wu L, Liu B, Yao J, et al. Ivonescimab Versus Pembrolizumab for Pd-L1-Positive Non-Small Cell Lung Cancer (Harmoni-2): A Randomised, Double-Blind, Phase 3 Study in China. *Lancet* (2025) 405(10481):839-49. Epub 2025/03/09. doi: 10.1016/S0140-6736(24)02722-3.

2. Wang L, Luo Y, Ren S, Zhang Z, Xiong A, Su C, et al. A Phase 1b Study of Ivonescimab, a Programmed Cell Death Protein-1 and Vascular Endothelial Growth Factor Bispecific Antibody, as First- or Second-Line Therapy for Advanced or Metastatic Immunotherapy-Naive Nsclc. *J Thorac Oncol* (2024) 19(3):465-75. Epub 2023/10/26. doi: 10.1016/j.jtho.2023.10.014.

3. Dhillon S. Ivonescimab: First Approval. *Drugs* (2024) 84(9):1135-42. Epub 2024/07/29. doi: 10.1007/s40265-024-02073-w.

4. Zak J, Pratumchai I, Marro BS, Marquardt KL, Zavareh RB, Lairson LL, et al. Jak Inhibition Enhances Checkpoint Blockade Immunotherapy in Patients with Hodgkin Lymphoma. *Science* (2024) 384(6702):eade8520. Epub 2024/06/20. doi: 10.1126/science.ade8520.

5. Bachanova V, Zak J, Cao Q, Maakaron J, Grzywacz B, Fletcher C, et al. Phase 1 Trial of Ruxolitinib Combined with Nivolumab in Patients with Relapsed/Refractory Hodgkin Lymphoma after Failure of Checkpoint Inhibitor (Cpi). *Hematological Oncology* (2023) 41(S2):582-. doi: 10.1002/hon.3164_436.

6. Gillessen S, Pluetschow A, Vucinic V, Ostermann H, Kobe C, Brockelmann PJ, et al. Jak Inhibition with Ruxolitinib in Relapsed or Refractory Classical Hodgkin Lymphoma: Final Results of a Phase Ii, Open Label, Multicentre Clinical Trial (Jericho). *Eur J Haematol* (2022) 109(6):728-35. Epub 2022/09/04. doi: 10.1111/ejh.13859.

7. Fong L, Hotson A, Powderly JD, Sznol M, Heist RS, Choueiri TK, et al. Adenosine 2a Receptor Blockade as an Immunotherapy for Treatment-Refractory Renal Cell Cancer. *Cancer Discov* (2020) 10(1):40-53. Epub 2019/11/17. doi: 10.1158/2159-8290.CD-19-0980.

8. Gomez-Roca C, Cassier P, Zamarin D, Machiels JP, Perez Gracia JL, Stephen Hodi F, et al. Anti-Csf-1r Emactuzumab in Combination with Anti-Pd-L1 Atezolizumab in Advanced Solid Tumor Patients Naive or Experienced for Immune Checkpoint Blockade. *J Immunother Cancer* (2022) 10(5). Epub 2022/05/17. doi: 10.1136/jitc-2021-004076.

9. Johnson M, Dudek AZ, Sukari A, Call J, Kunk PR, Lewis K, et al. Arry-382 in Combination with Pembrolizumab in Patients with Advanced Solid Tumors: Results from a Phase 1b/2 Study. *Clin Cancer Res* (2022) 28(12):2517-26. Epub 2022/03/19. doi: 10.1158/1078-0432.CCR-21-3009.

10. Sachdev J, Hu-Lieskovan S, Patnaik A, Eisenberg P, Ribas A. Phase 1/2a Study of Double Immune Suppression Blockade by Combining a Csf1r Inhibitor (Pexidartinib/Plx3397) with an Anti–Pd-1 Antibody (Pembrolizumab) to Treat Advanced Melanoma and Other Solid Tumors. *Gynecologic Oncology* (2016) 141(Suppl 1):147-8. doi: 10.1016/j.ygyno.2016.04.385.

11. Hong DS, Postow M, Chmielowski B, Sullivan R, Patnaik A, Cohen EEW, et al. Eganelisib, a First-in-Class Pi3kgamma Inhibitor, in Patients with Advanced Solid Tumors: Results of the Phase 1/1b Mario-1 Trial. *Clin Cancer Res* (2023) 29(12):2210-9. Epub 2023/04/01. doi: 10.1158/1078-0432.CCR-22-3313.

12. Armstrong AJ, Geva R, Chung HC, Lemech C, Miller WH, Jr., Hansen AR, et al. Cxcr2 Antagonist Navarixin in Combination with Pembrolizumab in Select Advanced Solid Tumors: A Phase 2 Randomized Trial. *Invest New Drugs* (2024) 42(1):145-59. Epub 2024/02/07. doi: 10.1007/s10637-023-01410-2.

13. Agarwal N, Azad A, Carles J. Contact-02: Phase 3 Study of Cabozantinib (C) Plus Atezolizumab (a) Vs Second Novel Hormonal Therapy (Nht) in Patients (Pts) with Metastatic Castration-Resistant Prostate Cancer (Mcrpc). *Journal of Clinical Oncology* (2024) 42(4_suppl):18-. doi: 10.1200/JCO.2024.42.4_suppl.18.

14. Agarwal N. Lba67 Cabozantinib (C) Plus Atezolizumab (a) Versus 2nd Novel Hormonal Therapy (Nht) in Patients (Pts) with Metastatic Castration-Resistant Prostate Cancer (Mcrpc): Final Overall Survival (Os) Results of the Phase Iii, Randomized, Contact-02 Study. *Annals of Oncology* (2024) 35(Suppl 2):S1256-S7. doi: 10.1016/s0923-7534(24)03890-0.

15. Wu S, Wu Z, Lu Z, Qi F, Cheng J, Chu T, et al. Selective Apoptosis of Tumor-Associated Platelets Boosts the Anti-Metastatic Potency of Pd-1 Blockade Therapy. *Cell Rep Med* (2025) 6(3):101984. Epub 2025/03/01. doi: 10.1016/j.xcrm.2025.101984.
