# Supplementary material for: Beyond monoclonal antibodies: constraints and the case for alternative PD-1/PD-L1-targeting formats
Source: Front Immunol. 2025 Dec 17;16:1729468. doi: 10.3389/fimmu.2025.1729468 (PMC12753384; doi:10.3389/fimmu.2025.1729468)
Supplement: Supplementary file 7 [file Table7.docx]

**Supplementary Table S7.** Comparative features of PD-1/PD-L1-targeting scaffolds (2025 update). Summary of properties across six modality classes; examples are illustrative rather than exhaustive

| Parameter | mAbs | BsAbs | sdAb (VHH “nanobodies”; sdAb-Fc) | Peptides / macrocycles | Small molecules | Decoys / aptamers |
| --- | --- | --- | --- | --- | --- | --- |
| Mechanism of action | Direct PD-1/PD-L1 blockade | Dual blockade / co-stimulation (e.g., PD-1×CTLA-4; PD-L1×4-1BB) | PD-(L)1 blockade (often as sdAb-Fc) | PD-(L)1 blockade; in some cases PD-L1 degradation (peptide-PROTAC) | Binding to PD-L1 pocket with dimerization and/or internalisation / degradation of PD-L1 | Sequestration of PD-(L)1 by decoys (PD-1–Fc); antagonism by aptamers |
| Molecular mass (order) | ~150 kDa | ~150–200 kDa | ~12–80 kDa (sdAb 12–15 kDa; sdAb-Fc higher) | ~1–3 kDa (macrocycles) | ~0.3–1 kDa | decoys ~150 kDa (Fc fusions); aptamers ~8–25 kDa  (+ modifications) |
| Route of administration | i.v./s.c. | i.v. | s.c./i.v.; gene-encoded/local options | Mostly parenteral; sometimes local | Oral (target); local | Parenteral; local/platform-based |
| Half-life & controllability | Long (2–3 weeks) | Long | Short (sdAb) to long (sdAb-Fc) | Short–moderate (improved by cyclisation/stapling) | Short (hours–days); rapid on/off control | Decoys - long; aptamers - short (need protection/size increase) |
| Tissue penetration | Limited | Limited / context-improved | Better than IgG | Higher than IgG | High | Variable; improved by conjugation/carriers |
| Immunogenicity (ADA) | Possible | Possible | Reduced with humanization | Low–moderate (chemistry-dependent) | No ADA; possible off-target pharmacology | Low (aptamers) / IgG-like for decoys |
| Manufacturing / cost | High (bioproduction) | High | Lower than IgG (esp. sdAb) | Moderate; specialised synthesis | Lower; scalable | Aptamers - low; decoys - IgG-like |
| Clinical stage (2025) | Widely used | Late-phase / first approvals (China) | Envafolimab (KN035) approved (China) | Early (imaging / preclinical therapeutics) | Early clinical (FiH / phase I) | Early / preclinical |
| Exemplars | Nivolumab, Pembrolizumab, Atezolizumab | Cadonilimab, KN046, GEN1046 | Envafolimab (KN035) | pAC65; [18F]BMS-986229 (PET tracer) | BMS-202 (tool), evixapodlin (GS-4224), INCB086550, MAX-10181 | AMP-224 (PD-L2–Fc), PD1-Fc; PD-L1 aptamers |
| Strengths | High affinity/selectivity | Dual-target logic; conditional agonism | s.c. dosing; compactness; better diffusion | Address large PPIs; modular | Oral potential; deep diffusion; fine medicinal-chemistry tuning | Modular chemistry; no ADA (aptamers) |
| Limitations | Cost; penetration; immunogenicity | Co-expression biomarkers; dosing complexity | T½ / residual VHH immunogenicity (mitigated by humanization) | Poor oral bioavailability; stability; species differences | PPI challenge; off-targets; need for stringent on-target validation | Sparse clinical efficacy signals; PK/stability (aptamers) |
| Key recent sources (2024–2025) | (1) | (2) | (3) | (4, 5) | (6-7) | (8-9) |

**Abbreviations:** ADA, anti-drug antibodies; BsAb, bispecific antibody; sdAb, single-domain antibody; VHH, variable domain of heavy-chain-only antibody; Fc, fragment crystallisable domain; FiH, first-in-human; i.v., intravenous; s.c., subcutaneous; PET, positron-emission tomography; PPI, protein–protein interaction; PK, pharmacokinetics; PD, pharmacodynamics; T½, terminal half-life.

**Note:** Exemplars are illustrative, not exhaustive; regulatory status refers to oncology indications as of 2025.

**References:**

1. Chan P, Liu SN, Gosselin N, Sauve Z, Marchand M, Lin A, et al. Population Pharmacokinetics and Exposure-Response of Subcutaneous Atezolizumab in Patients with Non-Small Cell Lung Cancer. *CPT Pharmacometrics Syst Pharmacol* (2025) 14(4):726-37. Epub 2025/02/05. doi: 10.1002/psp4.13310.

2. Zhao Y, Chen G, Li X, Wu J, Chang B, Hu S, et al. Kn046, a Bispecific Antibody against Pd-L1 and Ctla-4, Plus Chemotherapy as First-Line Treatment for Metastatic Nsclc: A Multicenter Phase 2 Trial. *Cell Rep Med* (2024) 5(3):101470. Epub 2024/03/21. doi: 10.1016/j.xcrm.2024.101470.

3. Cui C, Wang J, Wang C, Xu T, Qin L, Xiao S, et al. Model-Informed Drug Development of Envafolimab, a Subcutaneously Injectable Pd-L1 Antibody, in Patients with Advanced Solid Tumors. *Oncologist* (2024) 29(9):e1189-e200. Epub 2024/07/10. doi: 10.1093/oncolo/oyae102.

4. Mukherjee S, Rogers A, Creech G, Hang C, Ramirez A, Dummeldinger M, et al. Process Development of a Macrocyclic Peptide Inhibitor of Pd-L1. *J Org Chem* (2024) 89(10):6651-63. Epub 2024/04/25. doi: 10.1021/acs.joc.4c00430.

5. Armstrong AJ, Geva R, Chung HC, Lemech C, Miller WH, Jr., Hansen AR, et al. Cxcr2 Antagonist Navarixin in Combination with Pembrolizumab in Select Advanced Solid Tumors: A Phase 2 Randomized Trial. *Invest New Drugs* (2024) 42(1):145-59. Epub 2024/02/07. doi: 10.1007/s10637-023-01410-2.

6. Hec-Galazka A, Tyrcha U, Barczynski J, Bielski P, Mikitiuk M, Gudz GP, et al. Nonsymmetrically Substituted 1,1'-Biphenyl-Based Small Molecule Inhibitors of the Pd-1/Pd-L1 Interaction. *ACS Med Chem Lett* (2024) 15(6):828-36. Epub 2024/06/19. doi: 10.1021/acsmedchemlett.4c00042.

7. Cheng B, Lv J, Xiao Y, Song C, Chen J, Shao C. Small Molecule Inhibitors Targeting Pd-L1, Ctla4, Vista, Tim-3, and Lag3 for Cancer Immunotherapy (2020-2024). *Eur J Med Chem* (2025) 283:117141. Epub 2024/12/10. doi: 10.1016/j.ejmech.2024.117141.

8. Izadi S, Abrantes R, Gumpelmair S, Kunnummel V, Duarte HO, Steinberger P, et al. An Engineered Pd1-Fc Fusion Produced in N. Benthamiana Plants Efficiently Blocks Pd1/Pdl1 Interaction. *Plant Cell Rep* (2025) 44(4):80. Epub 2025/03/23. doi: 10.1007/s00299-025-03475-0.

9. Mohd Nazri MN, Khairil Anwar NA, Mohd Zaidi NF, Fadzli Mustaffa KM, Mokhtar NF. Pd-L1 DNA Aptamers Isolated from Agarose-Bead Selex. *Bioorg Med Chem Lett* (2024) 112:129943. Epub 2024/09/03. doi: 10.1016/j.bmcl.2024.129943.
