# Supplementary material for: Beyond monoclonal antibodies: constraints and the case for alternative PD-1/PD-L1-targeting formats
Source: Front Immunol. 2025 Dec 17;16:1729468. doi: 10.3389/fimmu.2025.1729468 (PMC12753384; doi:10.3389/fimmu.2025.1729468)
Supplement: Supplementary file 8 [file Table8.docx]

**Supplementary Table S8.** Near‑term shortlist and milestones for PD‑1/PD‑L1‑modulating scaffolds (2025–2026)

| Class | Representatives | What stands out | Evidence level / status (2025) | Biomarker prerequisites | Red flags (dose/toxicity/ops) | Near-term milestones (2025–2026) | Key sources (primary/regulatory/registry) |
| --- | --- | --- | --- | --- | --- | --- | --- |
| Bispecific antibodies | Cadonilimab (PD-1×CTLA-4); KN046 (PD-L1×CTLA-4); GEN1046 / acasunlimab (PD-L1×4-1BB) | Dual checkpoint blockade or conditional co‑stimulation to deepen responses | Late phase for class; China labels for cadonilimab; KN046 multiple phase‑2; GEN1046 phase‑1/2 | Co‑expression / immune contexture; for 4‑1BB agonism – PD‑L1 in TME | Dosing window; irAE management; costimulation‑related liver/GI risk | China label expansion (cervical) for cadonilimab; KN046 phase‑2 readouts; GEN1046 expansion cohort OS/DoR updates | Cadonilimab: NMPA label (2022; 2025 updates); KN046: late‑phase publications; GEN1046: NCT03917381 |
| sdAb (VHH) / sdAb‑Fc | Envafolimab (KN035) | Subcutaneous dosing; compact scaffold with validated PD‑L1 blockade | Approved in China; broader clinical optimisation (PK/PD, combinations) | PD‑L1 expression (context‑dependent); perioperative/combination context TBD | External generalisability; combination safety | Global expansion studies; potential OS/PFS updates 2025–2026 | China label/updates; sponsor communications; phase‑3 initiation notes |
| Small molecules | Evixapodlin (GS‑4224); INCB086550 | Oral PD‑L1 ligands with measurable human PD (ligand occupancy / internalisation) | First‑in‑human with PD biomarkers; additional phase‑1 cohorts | PD‑L1‑high target engagement; peripheral PD readouts | PPI challenge; off‑targets; need for stringent on‑target validation | Mature dose‑finding PK/PD; initial ORR/DoR signals in biomarker‑enriched cohorts | (1-3) |
| Peptides / macrocycles | BMS‑986189 (macrocycle); [18F]BMS‑986229 (PD‑L1 PET) | Manufacturing readiness (macrocycle); clinical imaging readouts (PET) | Therapeutic peptides preclinical; PD‑L1 PET in clinical use cases | Imaging: PD‑L1‑positive lesions; histology‑specific validation | Peptide oral bioavailability/stability; cross‑species affinity | Additional clinical PET datasets and correlation with response to PD‑(L)1 | (4) |
| Decoys / aptamers | PD‑1–Fc (plant‑produced); AMP‑224 (PD‑L2–Fc) | Non‑antibody route; modular manufacturability | Predominantly preclinical/early clinical tools; mixed clinical signals for AMP‑224 | None established; exploratory | PK/stability (aptamers); translational gaps for decoys | IND‑enabling studies for improved Fc/half‑life; limited pilot trials | (5, 6) |

**Abbreviations:** FiH, first‑in‑human; LOE, level of evidence; irAE, immune‑related adverse event; PD, pharmacodynamics; DoR, duration of response; OS, overall survival; PFS, progression‑free survival; TME, tumour microenvironment; PPI, protein–protein interaction; GI, gastrointestinal.

**Notes:** Evidence level reflects publicly available late‑phase trials, labels and registry records as of 6 October 2025 (JST). “Near‑term milestones” are indicative and depend on sponsor disclosures and conference schedules.

**References:**

1. Odegard JM, Othman AA, Lin KW, Wang AY, Nazareno J, Yoon OK, et al. Oral Pd-L1 Inhibitor Gs-4224 Selectively Engages Pd-L1 High Cells and Elicits Pharmacodynamic Responses in Patients with Advanced Solid Tumors. *J Immunother Cancer* (2024) 12(4). Epub 2024/04/12. doi: 10.1136/jitc-2023-008547.

2. Cheng B, Lv J, Xiao Y, Song C, Chen J, Shao C. Small Molecule Inhibitors Targeting Pd-L1, Ctla4, Vista, Tim-3, and Lag3 for Cancer Immunotherapy (2020-2024). *Eur J Med Chem* (2025) 283:117141. Epub 2024/12/10. doi: 10.1016/j.ejmech.2024.117141.

3. Hec-Galazka A, Tyrcha U, Barczynski J, Bielski P, Mikitiuk M, Gudz GP, et al. Nonsymmetrically Substituted 1,1'-Biphenyl-Based Small Molecule Inhibitors of the Pd-1/Pd-L1 Interaction. *ACS Med Chem Lett* (2024) 15(6):828-36. Epub 2024/06/19. doi: 10.1021/acsmedchemlett.4c00042.

4. Huang W, Son MH, Ha LN, Kang L, Cai W. Challenges Coexist with Opportunities: Development of a Macrocyclic Peptide Pet Radioligand for Pd-L1. *Eur J Nucl Med Mol Imaging* (2024) 51(6):1574-7. Epub 2024/03/16 21:47. doi: 10.1007/s00259-024-06680-3.

5. Izadi S, Abrantes R, Gumpelmair S, Kunnummel V, Duarte HO, Steinberger P, et al. An Engineered Pd1-Fc Fusion Produced in N. Benthamiana Plants Efficiently Blocks Pd1/Pdl1 Interaction. *Plant Cell Rep* (2025) 44(4):80. Epub 2025/03/23. doi: 10.1007/s00299-025-03475-0.

6. Mohd Nazri MN, Khairil Anwar NA, Mohd Zaidi NF, Fadzli Mustaffa KM, Mokhtar NF. Pd-L1 DNA Aptamers Isolated from Agarose-Bead Selex. *Bioorg Med Chem Lett* (2024) 112:129943. Epub 2024/09/03. doi: 10.1016/j.bmcl.2024.129943.
